# Historical and current introgression in a Mesoamerican hummingbird species complex: a biogeographic perspective

**DOI:** 10.7717/peerj.1556

**Published:** 2016-01-12

**Authors:** Rosa Alicia Jiménez, Juan Francisco Ornelas

**Affiliations:** Departamento de Biología Evolutiva, Instituto de Ecología A.C., Xalapa, Veracruz, Mexico

**Keywords:** Hummingbirds, Pleistocene, Mesoamerica, Phylogeography, *Amazilia*, Speciation, Introgression, Biogeography, Genetics, Ecology, Evolutionary studies

## Abstract

The influence of geologic and Pleistocene glacial cycles might result in morphological and genetic complex scenarios in the biota of the Mesoamerican region. We tested whether berylline, blue-tailed and steely-blue hummingbirds, *Amazilia beryllina*, *Amazilia cyanura* and *Amazilia saucerottei*, show evidence of historical or current introgression as their plumage colour variation might suggest. We also analysed the role of past and present climatic events in promoting genetic introgression and species diversification. We collected mitochondrial DNA (mtDNA) sequence data and microsatellite loci scores for populations throughout the range of the three *Amazilia* species, as well as morphological and ecological data. Haplotype network, Bayesian phylogenetic and divergence time inference, historical demography, palaeodistribution modelling, and niche divergence tests were used to reconstruct the evolutionary history of this *Amazilia* species complex. An isolation-with-migration coalescent model and Bayesian assignment analysis were assessed to determine historical introgression and current genetic admixture. mtDNA haplotypes were geographically unstructured, with haplotypes from disparate areas interdispersed on a shallow tree and an unresolved haplotype network. Assignment analysis of the nuclear genome (nuDNA) supported three genetic groups with signs of genetic admixture, corresponding to: (1) *A. beryllina* populations located west of the Isthmus of Tehuantepec; (2) *A. cyanura* populations between the Isthmus of Tehuantepec and the Nicaraguan Depression (Nuclear Central America); and (3) *A. saucerottei* populations southeast of the Nicaraguan Depression. Gene flow and divergence time estimates, and demographic and palaeodistribution patterns suggest an evolutionary history of introgression mediated by Quaternary climatic fluctuations. High levels of gene flow were indicated by mtDNA and asymmetrical isolation-with-migration, whereas the microsatellite analyses found evidence for three genetic clusters with distributions corresponding to isolation by the Isthmus of Tehuantepec and the Nicaraguan Depression and signs of admixture. Historical levels of migration between genetically distinct groups estimated using microsatellites were higher than contemporary levels of migration. These results support the scenario of secondary contact and range contact during the glacial periods of the Pleistocene and strongly imply that the high levels of structure currently observed are a consequence of the limited dispersal of these hummingbirds across the isthmus and depression barriers.

## Introduction

Current studies of molecular biogeography employ several genetic markers in both the nuclear and mitochondrial genome, with broad taxon sampling encompassing a broad geographic scope. Within species, the resulting patterns of phylogeographic breaks that arise between mtDNA clades generally align with those observed in the nuclear genome (Avise, 1994; Zink & Barrowclough, 2008). However, concordant patterns between mtDNA and nuDNA are not always observed (Funk & Omland, 2003; Toews & Brelsford, 2012). Discordance between mtDNA and nuDNA (hereafter mito-nuclear discordance; Toews & Brelsford, 2012) is expected because the mitochondrial genome is haploid and typically uniparentally inherited and, therefore, has a fourfold smaller effective population size (Zink & Barrowclough, 2008; Toews & Brelsford, 2012), completing the process of lineage divergence (i.e., ancestral polymorphisms are lost over time) faster than nuDNA. Despite the use of numerous nuclear loci, mito-nuclear discordance can also arise if there are differences in how selection acts on the mitochondrial genome as compared to the nuclear genome or if there is biased movement of either marker type driven by demographic asymmetries such as sex-biased dispersal (Rheindt & Edwards, 2011; Toews & Brelsford, 2012). Distinguishing between incomplete lineage sorting and these other types of discordance can be difficult (McKay & Zink, 2010). However, the mito-nuclear discordance that arises from incomplete lineage sorting is not expected to leave any predictable phylogeographic pattern (Funk & Omland, 2003; Toews & Brelsford, 2012); if strong geographic inconsistencies between patterns in mtDNA and nuDNA are observed, incomplete lineage sorting can be ruled out as an explanation for the mito-nuclear discordance.

Most of the taxa that display patterns of biogeographical mito-nuclear discordance are groups of closely related species that were isolated for long periods of time and are either currently in secondary contact, or have experienced range contact at some point in their past. Upon secondary contact, these groups formed hybrid zones, interbreeding to varying extents, and the mito-nuclear discordance was promoted by divergent patterns of gene flow between the two genomes (Toews & Brelsford, 2012). Potential range expansions and contractions in response to Pleistocene climate cycles may have contributed to the historical isolation and secondary contact of these taxa (Hewitt, 2000; Zamudio & Savage, 2003; Melo-Ferreira et al., 2012; Campagna et al., 2014; Giarla & Jansa, 2015; Komaki et al., 2015; Ornelas et al., 2015). The resulting distribution dynamics have been suggested as the primary cause of historical mtDNA introgression events (Mao et al., 2013; Rodríguez-Gómez & Ornelas, 2015), which might result in complex morphological and genetic scenarios (Rheindt & Edwards, 2011; Acevedo et al., 2015). Climatic fluctuations can increase or decrease the available habitat for a species, having important consequences on its population size (Malpica & Ornelas, 2014; Giarla & Jansa, 2015). An increase in population size and range expansion might promote secondary contact between two different populations leading to hybridization and genetic interchange (Zamudio & Savage, 2003; Campagna et al., 2014), whereas a decrease in population size and range contraction can isolate populations and promote allopatric speciation (Wiens, 2004; Wiens & Graham, 2005). Deciphering the resulting complex demographic scenarios is a first step to understanding the role of historical and current factors in shaping biodiversity in a particular region.

Mesoamerica, a region located from Mexico to northern Costa Rica, is among the richest biodiversity hotspots in the world (Myers et al., 2000; Mittermeier et al., 2005). Mesoamerican biodiversity is the result of biotic interchange between North America and South America (e.g., Weir, Bermingham & Schluter, 2009; Smith & Klicka, 2010; Ornelas et al., 2014) and autochthonous diversification linked to its complex relief, diversity of habitats and dynamic tectonic and climate history (Barrier et al., 1998; Manea et al., 2005; Rovito et al., 2015). The distribution and composition of the Mesoamerican biota have been strongly influenced by geological and climatic events, with considerable invasions of Mesoamerica by temperate elements from North America and South American tropical elements prior to the formation of the Isthmus of Panama c. 4.5 Ma (Raven & Axelrod, 1974; Daza, Castoe & Parkinson, 2010; Ornelas et al., 2014), and fragmentation of the extensive tropical forest with species restricted to refuge populations in Mesoamerica during the glacial maxima of the Pleistocene (1.6–0.01 Ma) (Hewitt, 2000). The resulting biogeographical patterns reflecting the significant influence of dispersal and isolation, extinction and colonization on the populations of this region from the Miocene onward, however, remain contentious. Recent phylogenetic and biogeographical evidence has uncovered multiple dispersal and vicariant events in the region that occurred starting in the middle Miocene, prior to the formation of the Isthmus of Panama (Daza, Castoe & Parkinson, 2010; Ornelas et al., 2014; Bacon et al., 2015). Within Mesoamerica, continuous tectonic activity uplifted the highlands and repeated cycles of forest contraction and expansion in the highlands, owing to Pleistocene climate cycles, formed a set of corridors and barriers, creating further isolation and shaping genetic divergence and autochthonous diversification in the region at different time scales (e.g., Gutiérrez-García & Vázquez-Domínguez, 2012; Rodríguez-Gómez & Ornelas, 2014; Rovito et al., 2015). Several phylogeographical studies have shown marked genetic divergence between populations on either side of the Isthmus of Tehuantepec in southern Mexico, a common barrier of dry scrubby lowlands for many taxa (e.g., Bonaccorso et al., 2008; Barber & Klicka, 2010; Barrera-Guzmán et al., 2012; Ornelas et al., 2013; Ortiz-Ramírez et al., 2016), including hummingbirds (Cortés-Rodríguez et al., 2008; González, Ornelas & Gutiérrez-Rodríguez, 2011; Arbeláez-Cortés & Navarro-Sigüenza, 2013; Rodríguez-Gómez, Gutiérrez-Rodríguez & Ornelas, 2013; Zamudio-Beltrán & Hernández-Baños, 2015; Rodríguez-Gómez & Ornelas, 2015), and between populations separated by the Nicaraguan Depression, a lowland corridor running from the Caribbean to the Pacific near the border between Costa Rica and Nicaragua (Bonaccorso et al., 2008; Zamudio-Beltrán & Hernández-Baños, 2015; Ortiz-Ramírez et al., 2016). However, these geographic barriers seem permeable for highland species during the colder stages of the glacial cycles (Gutiérrez-Rodríguez, Ornelas & Rodríguez-Gómez, 2011; Rodríguez-Gómez, Gutiérrez-Rodríguez & Ornelas, 2013; Rodríguez-Gómez & Ornelas, 2014; Ornelas & Rodríguez-Gómez, 2015). Using phylogeographical data and species distribution modelling, Ornelas et al. (2015) described the various demographic responses of eight hummingbird species to Pleistocene climate changes in the region, including postglacial population expansion to northern areas, elevation isolation in the highlands of southern Chiapas and Guatemala, population expansion and range shifts through sites with suitable habitats or no demographic changes with persistence across the geographical range throughout glacial cycles, suggesting that the responses of these hummingbirds are idiosyncratic. The populations of several species show both marked genetic and morphological breaks related to these barriers (e.g., Barrera-Guzmán et al., 2012; Zamudio-Beltrán & Hernández-Baños, 2015). However, species with complex patterns of morphological variation including intermediate phenotypes and widespread sharing of haplotypes across the barriers suggesting incomplete lineage sorting and genetic introgression have been rarely investigated (Milá et al., 2011).

**Figure 1:**
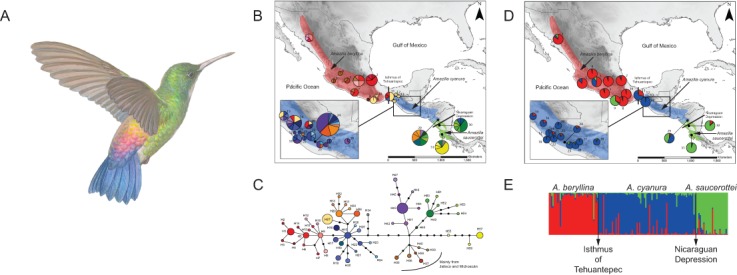
Distribution ranges and genetic differentiation among *Amazilia beryllina*, *A. saucerottei* and *A. cyanura* hummingbirds. (A) The blue-tailed hummingbird (*Amazilia cyanura*), a sexually monochromatic species characteristic of tropical lowland forests in Central America. Drawing by Marco Pineda (courtesy of Juan Francisco Ornelas). (B) Approximate range map of *beryllina*, *cyanura* and *saucerottei* and haplotype geographic distribution. Pie charts represent haplotypes found in each sampling locality. Section size in the pie charts corresponds to the number of individuals with that haplotype. Numbers next to the pie charts refer to localities as those used in Table S1. (C) Haplotype network of mitochondrial DNA (*ND2* and *ATPase 6–8*) of three *Amazilia* species. (D) Geographical distribution of three clusters according to STRUCTURE v. 2.3.4 and composition of the genetic cluster in each population. Three individuals from populations 11, 12 and 17 were not included because of amplification problems. (E) Bar plot showing Bayesian assignment probabilities from the software STRUCTURE for *K* = 3. Each vertical bar corresponds to one hummingbird. The proportion of each bar that is red, blue, and green represents an individual’s assignment probability to clusters one, two, and three, respectively. Individuals are grouped into sampling localities and listed from left to right from northern Mexico to Central America along the x-axis.

Hummingbirds provide a unique research opportunity for assessing the relative importance of genetic introgression during the initial stages of species separation (Rheindt & Edwards, 2011), because the distributions of closely related species of several hummingbird groups overlap extensively (Schuchmann, 1999), closely related species that overlap this way can easily hybridize (Abbott et al., 2013), and this clade has among the highest rates of hybridization (Grant & Grant, 1992; McCarthy, 2006). Among hummingbird genera, *Amazilia* is exceptionally rich (c. 29 species) with species distributed from the southern USA to southern Brazil (Ornelas et al., 2014). The distributions of several *Amazilia* species overlap extensively, and the clade has a high rate of hybridization, between *Amazilia* species and between *Amazilia* species and those of other genera (e.g., *Chrysuronia*, *Cynanthus*, *Eugenes*, and *Hylocharis*; McCarthy, 2006). Three species of *Amazilia* hummingbirds, *A. beryllina* (Deppe), *A. cyanura* Gould, *A. saucerottei* (DeLattre and Boucier), that show some overlap in their distributions, have extensive plumage colour variation that has not been fully explained, and there are reports of hybridization. The taxonomic history of the species complex is mired in problems. *Amazilia beryllina* populations occur in montane habitats from north-western Mexico (rarely seen in the south-western US) to central Honduras where five subspecies (*A. b. viola* in the Sierra Madre Occidental from SE Sonora to Michoacán and Guerrero, *A. b. beryllina* in central Mexico from Estado de México to S Veracruz and Oaxaca, *A. b. lichtensteini* from western Chiapas, *A. b. sumichrasti* from central and southern Chiapas, and *A. b. devillei* from southern Guatemala and El Salvador to central Honduras) have been described based on slight differences in morphology and distribution; *Amazilia cyanura* (Fig. 1A) populations occupy mid-elevation habitats from south-eastern Chiapas, Mexico to Costa Rica, where three subspecies were named based on geography (*A. c. guatemalae* in SE Chiapas and S Guatemala, *A. c. cyanura* in S Honduras, east of El Salvador and NW Nicaragua, and *A. c. impatiens* in Costa Rica); and *Amazilia saucerottei* populations occur at low elevation habitats from western Nicaragua to Costa Rica, Colombia and Venezuela, where four subspecies (*A. s. hoffmanni* in W Nicaragua to Costa Rica, *A. s. warscewiczi* in the N and C of Colombia and NW Venezuela, *A. s. saucerottei* in W Colombia, and *A. s. braccata* in the Andes of W Venezuela) are currently recognized based on geography (Fig. 1B; Schuchmann, 1999; Dickinson & Remsen, 2013). The distributional ranges of *beryllina* and* cyanura* overlap in some areas of Chiapas, Guatemala, El Salvador, and Honduras (Howell & Webb, 1995; Schuchmann, 1999), and the distributional ranges of *cyanura* and *saucerottei* overlap in Nicaragua.

Recent molecular work based on mtDNA sequences suggests monophyly of *beryllina* and *cyanura* from northern Mesoamerica, diverging at the Pleistocene (2.83–1.09 million years ago). However, species delimitation remained unresolved because the one sample of *cyanura* is nested within a clade of several *beryllina* individuals (Ornelas et al., 2014). These two species differ mainly in tail colouration, however, natural hybridization between these two species cannot be ruled out based on the intermediate colouration of a specimen from Guatemala that was first noted by Ridgway (1911) and tail colour variation in specimens from El Salvador that were observed later by Dickey & van Rossem (1938). Since then, several authors have referred to this phenotypic variation as potentially interspecific hybridization (e.g., Howell & Webb, 1995; McCarthy, 2006), or that individual differences in tail colouration might be due to intraspecific phenotypic variation in *beryllina* (Schuchmann, 1999). These studies did not consider saucerottei as part of this species complex. The mtDNA phylogeny of *Amazilia* presented by Ornelas et al. (2014) includes one sample of *saucerottei* from Mérida, Venezuela nested within a different clade of *Amazilia* species composed of *A. tobaci* (Trinidad and Tobago), *A. viridigaster* (Venezuela, Amazonas) and *A. edward* (Panama). *Amazilia cyanura* and *A. saucerottei*, sympatric in Nicaragua, differ solely in the rufous wing patch in *cyanura* (Schuchmann, 1999), and those with the *saucerottei* phenotype are found in Guatemala where the species has not been previously reported; no one has considered that Central American *saucerottei* interbreed with *cyanura* in areas of sympatry. The time-calibrated phylogeny of McGuire et al. (2014) using both nuclear and mtDNA sequence data for 284 hummingbird species showed that the *saucerottei* sample from Central America (*A. saucerottei hoffmanni*, FMNH 394217) is more closely related to *beryllina* and *cyanura* than to its two conspecifics from South America (JPLV34, JPLV60), which are more closely related to other *Amazilia* species as suggested by Ornelas et al. (2014). Although Central American *A. saucerottei hoffmanni* resemble those *saucerottei* from South America (Schuchmann, 1999), the molecular evidence suggesting they belong to a different group of *Amazilia* species (e.g., McGuire et al., 2014) supports the proposal of Stiles & Skutch (1989) in considering the Central American populations of *saucerottei* as a different species (*A. sophiae*) based on notorious behavioural and vocal differences. However, the unresolved relationships within the group of *A. beryllina*, *A. cyanura* and Central American *A. saucerottei hoffmanni* syn. *A. sophiae* (Stiles & Skutch, 1989) suggest incomplete lineage sorting and hybridization among them, as these processes have been proposed as the major causes behind haplotype sharing between species (Rheindt & Edwards, 2011). In spite of this, vocal or behavioural differences between the three species (*A. beryllina*, *A. cyanura*, *A. saucerottei*), which could play a role in assortative mating and interspecific introgression, have not been quantified.

Here we examine the molecular biogeography of *beryllina*, *cyanura* and *saucerottei* hummingbird species to infer the role of Mesoamerican geographic barriers and recent glacial dynamics in driving the historical isolation and secondary contact of these taxa. We used mtDNA sequence data and microsatellite loci and extensive population-level sampling across the full range of these three *Amazilia* species, jointly analysed with morphological, ecological, and distributional data, to assess the following species divergence scenarios: (1) deep splits between species, in which the *Amazilia* species as taxonomically defined represent fully isolated clades defined by unique mtDNA haplotypes and representative microsatellite allele frequencies (mito-nuclear concordance); (2) the process of speciation is recent and occurred in the presence of gene flow, in which the following biogeographic mito-nuclear discordance patterns (Toews & Brelsford, 2012) are expected: (a) haplotype sharing of mtDNA between the three clades with no discernible geographic pattern in the distribution of the mtDNA clades (incomplete lineage sorting of mtDNA); (b) haplotype sharing maintained at low frequency across the range of the three taxa (complete introgression of mtDNA); (c) hummingbirds in the range of *cyanura* have mtDNA from *beryllina* and *saucerottei* at the range edges (partial introgression of mtDNA); or (d) a pattern of nuclear genetic divergence between lineages separated by the isthmus and depression barriers, with genetic signatures of secondary contact or range contact matching historic glacial events.

## Methods

### Field study permissions

Our fieldwork was conducted with the permission of Mexico’s Secretaría de Medio Ambiente y Recursos Naturales, Subsecretaría de Gestión para la Protección Ambiental, Dirección General de Vida Silvestre (permit numbers Dirección de Vida Silvestre, INE, SEMARNAP, D00-02/3269, SGPA/DGVS/07701/11) and the Consejo Nacional de Áreas Protegidas (CONAP) in Guatemala (permit number 14815). We thank the staff and owners of private reserves (San Jerónimo Miramar and Quixayá, Santa Rosa Sumatán, La Igualdad, Los Tarrales, Patrocinio, El Pilar, Cabaña Suiza, Nueva Granada, El Rosario, Irlanda), Municipalidad de Todos Santos Cuchumatán, and the Asociación de Reservas Naturales Privadas de Guatemala for allowing us to access these areas.

### Sampling and field procedures

For the molecular analysis, we sampled 154 hummingbirds from 31 localities between 2009 and 2013, covering most of the geographical range of *beryllina*, *cyanura* and *saucerottei* (Fig. 1B, Tables S1 and S2 in Supporting information). Hummingbirds were captured using mist nets, and two rectrices were collected from each hummingbird as a source of DNA for subsequent genetic analysis before the bird was released. Samples were collected under the required permits and using approved animal welfare protocols. Thirty-seven hummingbirds were collected and deposited as vouchers in museum collections (Escuela de Biología, USAC; Instituto de Ecología, A.C.). Our sampling was supplemented with 30 samples obtained on loan from tissue collections. The samples used cover the wide observed phenotypic variation, including the phenotypes of *beryllina*, *cyanura*, *saucerottei*, hummingbirds with intermediate colouration, and samples from both allopatric and sympatric ranges. Detailed information from museum specimens is provided in Tables S1 and S2.

### Mitochondrial DNA sequencing

Total genomic DNA was extracted using the DNeasy blood and tissue extraction kit (Qiagen, Valencia, CA, USA), following the protocol recommended by the manufacturer. Three mtDNA genes—350 base pairs (bp) of NADH nicotinamide dehydrogenase subunit 2 (*ND2*), and *ATPase 6* and *ATPase 8* (750 bp)—were amplified by polymerase chain reactions (PCR) and sequenced to infer phylogenetic relationships among haplotypes. Amplification of *ND2* was conducted with primers L5215 and H5578 (Hackett, 1996), whereas for the *ATPase 6–8* we used L8929 and H9855 (Sorenson et al., 1999). Protocols for PCR reactions and for sequencing the PCR products are described elsewhere (González, Ornelas & Gutiérrez-Rodrígue, 2011; Licona-Vera & Ornelas, 2014). Sequences were read in a 310 or 3730 automated DNA sequencer (Applied Biosystems, Foster City, CA, USA) at the Instituto de Ecología, A.C. sequencing facility, or at the Evolutionary Genetics Lab in the Museum of Vertebrate Zoology (MVZ).

### Summary statistics and haplotype network

Mitochondrial DNA sequences were assembled by eye and checked for stop codons using SEQUENCHER v. 4.9 (Genecodes, Ann Arbor, MI, USA) and then manually aligned with SE-AL v. 2.0a11 (http://tree.bio.ed.ac.uk/software/seal). All unique sequences used in this study are deposited in GenBank (Accession numbers KM198972–KM199267; Table S2).

Using DnaSP v. 4.2 (Rozas et al., 2003), the sequences of each region and combined sequences were examined for haplotype variation (*H*), segregating sites (*S*), haplotype diversity (*h*) and nucleotide diversity (*π*) per species. A statistical haplotype network was generated with the median-joining algorithm in NETWORK v. 4.6.1.1 (http://www.fluxus-technology.com) using the combined mtDNA matrix (Bandelt, Foster & Röhl, 1999) to visualize haplotype relationships.

### Phylogenetic analysis

A Bayesian inference (BI) phylogenetic tree was constructed using MRBAYES v. 3.1.2 (Huelsenbeck & Ronquist, 2011) with data partitioned by region (*tRNA*) and each codon position within the coding genes (*ND2*, *ATPase 6* and *ATPase 8*), as suggested by Bayes Factor analysis (Kass & Raftery, 1995). The substitution model for each partition was identified using jMODELTEST v. 0.1.1 (Posada, 2008) and the Akaike information criterion (AIC; Table S3).

Seven species of the emerald hummingbird group were included as outgroups, including conspecifics *A. saucerottei* from South America, *A. edward*, *A. tobaci*, *A. viridigaster*, *A. cyanocephala*, *A. violiceps*, *A. viridifrons*, and *Campylopterus curvipennis* based on McGuire et al. (2014) and Ornelas et al. (2014). Most of the sequences are new to this study; others were downloaded from GenBank (*A. saucerottei*, EU042523 and GU167205; *A. viridigaster*, EU042526).

The BI analysis employed partition-specific DNA evolution models for each gene with two parallel Markov chain Monte Carlo (MCMC) analyses executed simultaneously; each was run for 20 million generations, sampling every 1000 generations. A majority consensus tree was calculated, showing nodes with a posterior probability (PP) of 0.5 or more. Bayesian PP values were calculated from the sampled trees remaining after 5000 burn-in samples were discarded (Huelsenbeck & Ronquist, 2011) to only include trees after stationarity was reached. The consensus tree was visualized in FIGTREE v. 1.2.3 (http://tree.bio.ed.ac.uk/software/figtree/).

### Divergence time estimation

The time of the most recent common ancestor (TMRCA) of the clade *beryllina-cyanura-saucerottei* was estimated using BEAST v. 1.5.4 (Drummond & Rambaut, 2007). We used the mtDNA sequences partitioned by gene (*ND2* and *ATPase 6–8*) and those of the outgroup hummingbird species. The best-fit model of evolution, HKY+G for each partition, empirical base frequencies, and an uncorrelated lognormal relaxed model as the clock model were used. A coalescent model assuming constant population size was used to model the tree prior. The coalescent tree prior used in this analysis appears to be a better fit when mixed datasets are predominantly intraspecific data (Ho et al., 2011). We grouped the *Amazilia* sequences and constrained them to be monophyletic (Ornelas et al., 2014).

To calibrate the tree we used the substitution rate of 0.029 substitutions per site per million years (s/s/My; 0.0145 s/s/l/My) for the *ND2* and the geometric mean of 0.026 and 0.019 for the *ATPase 6–8* (0.0222 s/s/My; 0.0111 s/s/l/My) obtained for Hawaiian honeycreepers (Lerner et al., 2011), or the average rate of 0.0068 s/s/My (0.0034 s/s/l/My) for the *ND2* and the geometric mean of 0.0059 and 0.0047 (0.00265 s/s/l/My) for the *ATPase 6–8* obtained for major bird orders (Pacheco et al., 2011).

The root of the tree was calibrated using the average 27.7 Ma (normal prior, SD 3.5, range 35.4–19.9 Ma) divergence time for the basal split between *C. curvipennis* and all the *Amazilia* hummingbirds (Ornelas et al., 2014), followed by the age of the crown clade formed by *A. cyanocephala*, *A. beryllina*, *A. cyanura*, *A. edward*, *A. saucerottei*, *A. viridigaster*, *A. violiceps* and *A. viridifrons* (normal prior, mean 13.9 Ma, SD 3.0, range 19.8–8.0; Ornelas et al., 2014) as a secondary calibration.

We performed one run of 30 million generations with random starting trees, sampling every 1000 steps, and discarding the first 10% of trees as burn-in. Results were viewed in TRACER v. 1.5 (Drummond & Rambaut, 2007) to ensure that the effective sample sizes (ESS) for all priors and the posterior distribution were higher than 200, and finally annotated the trees using TREEANNOTATOR v. 1.5.4 (Drummond & Rambaut, 2007) summarized as a maximum clade credibility tree with mean divergence times and 95% highest posterior density (HPD) intervals of age estimates and visualized in FIGTREE.

### Historic population changes

The demographic history of each *Amazilia* species was inferred by means of neutrality tests and mismatch distributions with ARLEQUIN v. 3.5 (Excoffier & Lischer, 2010). To test whether populations evolved under neutrality, Fu’s *Fs* test and Tajima’s *D* tests were calculated with 1000 permutations, and mismatch distributions were calculated using the sudden expansion model of Schneider & Excoffier (1999) with 9000 bootstrap replicates. The validity of the sudden expansion assumption was determined using the sum of squared deviations (SSD) and Harpending’s raggedness index (Hri), which are higher in stable, non-expanding populations (Rogers & Harpending, 1992).

### Coalescent analysis

The Isolation-with-Migration model implemented in IMa (Hey & Nielsen, 2004, 2007) was used to determine whether mtDNA genetic divergence occurred in the presence of gene flow. Analyses of IMa were carried out between groups of populations using the combined data matrix, *ND2* and *ATPase 6–8*. We analysed mtDNA data as three pairwise comparisons in which hummingbirds were assigned to three genetic groups according to the results obtained using microsatellite data. For each group comparison we estimated the effective population size of the ancestral (*q*_A_) and the two descendant populations (*q*_1_ and *q*_2_), effective number of migrants per generation in both directions (*m*_1-to-2_ and *m*_2-to-1_), and time since divergence (*t*) at which the ancestral population gave rise to the descendant populations.

Three independent runs of 50 million generations were performed for each group comparison under the HKY model of evolution using the parameter values empirically determined in the preliminary runs to verify convergence of the different independent analyses. Each run used identical conditions, but different starting seed values, and a burn-in period of 30 million steps.

We used the same mean substitution rates for *ND2* and *ATPase 6–8* described above (Lerner et al., 2011; Pacheco et al., 2011) to estimate the effective population size (*Ne*) of each genetic group. The mutation rate was converted to per locus rate by multiplying the fragment length in base pairs for conversion to demographic units (Hey & Nielsen, 2007).

To convert the effective population size estimates, we used a 2-y generation time proposed for other *Amazilia* species (Rodríguez-Gómez, Gutiérrez-Rodríguez & Ornelas, 2013) based on the observation that the age of maturity begins one year after hatching, and an assumed low or high annual adult survival rates of 0.3 reported for *Colibri thalassinus* (Ruiz-Gutiérrez et al., 2012) and *Archilochus colubris* (Hilton & Miller, 2003) and 0.52 for an emerald resident species, *Hylocharis leucotis* (Ruiz-Gutiérrez et al., 2012). The approximate average generation time (T) is calculated according to T = *a* + (*s*/(1 − *s*)) (Lande, Engen & Sæther, 2003), where *a* is the time to maturity and *s* is the adult annual survival rate. Based on this, estimates for T range from 2.43 to 3.08 years.

To convert the time since divergence parameter of IMa to years, *t*, we divided the time parameter (*B*) by the mutation rate per year (*U*) converted to the per locus rate by multiplying by the fragment length in base pairs and, calculated this for the low and high rates (Lerner et al., 2011; Pacheco et al., 2011).

Because IMa assumes that there has not been any recombination within the genes that are being studied since the time of common ancestry of the gene copies included in the study (Hey & Nielsen, 2004, 2007), we conducted a recombination test using RDP v. 4.63 for the complete alignment of the mitochondrial sequences (Martin et al., 2010). We applied the methods RDP (Martin & Rybicki, 2000), MaxChi (Smith, 1992), and Chimaera (Posada & Crandall, 2001), with the *P*-value of 0.05. None of these methods detected any evidence of recombination in the alignment.

### Microsatellite genotyping

Samples from 145 hummingbirds were genotyped at twelve polymorphic, unlinked, nuclear microsatellite loci designed for *Amazilia cyanocephala* (A1-3-5, A1-4-1, A2-1-2 and A2-5-3; Molecular Ecology Resources Primer Development Consortium et al., 2013), *Campylopterus curvipennis* (Cacu1-10, Cacu4-8, Cacu13-1, Cacu13-7; Molecular Ecology Resources Primer Development Consortium et al., 2010) and *Selasphorus platycercus* (HumB1, HumB2, HumB3, HumB9; Oyler-McCance et al., 2011). Nine samples were not included because of amplification problems. PCR conditions and fragment sizing for microsatellite loci are fully described elsewhere (Molecular Ecology Resources Primer Development Consortium et al., 2010; Molecular Ecology Resources Primer Development Consortium et al., 2013; Oyler-McCance et al., 2011). Fluorescently labelled PCR fragments were read in a 310 or 3730 automated DNA sequencer (Applied Biosystems, Foster City, CA, USA) at the Instituto de Ecología, A.C. sequencing facility, or at the Evolutionary Genetics Lab in the MVZ. Alleles peaks were visualized using GENEMAPPER v. 4.1 (Applied Biosystems, Foster City, CA, USA) against an internal size standard (GeneScan-600 LIZ or 500 LIZ size standard; Applied Biosystems, Foster City, CA, USA) and scored manually.

### Population differentiation

The extent of linkage disequilibrium between pairs of loci, and departures from Hardy-Weinberg (H-W) equilibrium within populations and loci were calculated using GENEPOP v. 4.2 (Rousset, 2008) with Bonferroni corrections applied to correct for multiple simultaneous comparisons (Rice, 1989). Null allele frequencies for each locus were estimated using FreeNA (Chapuis & Estoup, 2007).

Population genetic structure based on Bayesian clustering was inferred using STRUCTURE v. 2.3.4 (Pritchard, Stephens & Donelly, 2000). We ran STRUCTURE under the admixture model with correlated allele frequencies without prior information on population origin. Ten independent chains were run for each *K*, from *K* = 1 to *K* = 8. The length of the burn-in was 100,000 and the number of MCMC iterations after burn-in was 500,000 and convergence was achieved. The most likely number of populations was determined estimating the DeltaK (Δ*K*) and the log likelihood of *K*, *Ln* P(*K*) = L(*K*) between successive *K* values (Evanno, Regnaut & Goudet, 2005). The identity of any individual as a putative migrant or migrant ancestry was assessed through the model with prior population information, assigning individuals in *K* populations, based on the DeltaK results. A migration rate of 0.05 was assumed. Finally, a mean-centred principal component analysis (PCA) on the microsatellite individual-genotype matrix was conducted using the R package *ADEgenet* (Jombart, 2008) to compare the clustering results with those obtained using STRUCTURE.

### Contemporary and historical migration rates

To compare migration rates over contemporary and historical timescales, we followed Chiucchi and Gibbs (2010) approach and analysed microsatellite data using the programs BAYESASS (Wilson & Rannala, 2003) and MIGRATE (http://popgen.sc.fsu.edu; Beerli, 2009), respectively. Using a Bayesian inference approach, BAYESASS estimates recent migration rates between populations within the last few generations (*m*), whereas MIGRATE uses the coalescent approach to jointly estimate the relative effective population size θN_e_ (4N_e_μ) and asymmetrical gene flow *M* (m/μ) between pairs of populations over much longer periods of time, approximately thousands of years (ca. 4N_e_ generations in the past; Beerli, 2009). We were able to use these software results as our microsatellite loci data proved to be unlinked (see Results). Briefly, BAYESASS was initially run with the default delta values for allelic frequency (*A*), migration rate (*M*), and inbreeding (*F*). Subsequent runs incorporated different delta values to ensure that the acceptance rate for proposed changes in parameters were between 20–40% for each parameter. Adjusted final delta values used were Δ*A* = 0.3 (35% acceptance rate), Δ*M* = 0.2 (27%), and Δ*F* = 0.3 (34%), respectively. To ensure convergence, we performed five independent runs (10 million iterations, 1 million burn-in, and sampling frequency of 1000) each with a different seed number, comparing the posterior mean parameter estimates for concordance. We also analysed the trace file of each run with TRACER to ensure an appropriate mixing of parameters and burn-in number. For this best fit run, we then ran the analysis increasing the run length to 30 million iterations with a 3-million burn-in.

We ran MIGRATE incorporating Bayesian inference analyses to estimate historical migration rates (*M*) among groups of populations. We used a Brownian-motion model with a constant mutation rate and *F*_ST_ to estimate θ. We performed several short runs to look for the appropriate priors. After finding suitable priors, MIGRATE was run three times to confirm convergence. These final runs consisted of one long chain, 1,000,000 sampled trees, 10,000 recorded, with a burn-in of 10,000 with 30 replicates and each run with a different seed number. We set the minimum and maximum boundaries for theta (θ) and migration (*M*) as 0.0 and 100.0, with a delta value of 10. A four-chain heating at temperatures of 1, 1.5, 3 and 10,000 was implemented to increase the efficiency of the MCMC (Chiucchi & Gibbs, 2010). We present results for *M* estimates from one randomly chosen run out of the three final runs as their parameter estimates were similar.

Lastly, we performed a Mantel test with 5000 permutations to test for similarity between contemporary and historical values of *m*. For this analysis, we used the values of *m* directly generated by BAYESASS and estimated *m* from values of *M* (m/μ) generated by MIGRATE by dividing all *M* values by an estimated mutation rate of 5 × 10^−4^ for microsatellites (Garza & Williamson, 2001).

### Morphological variation

Plumage colour variation was assessed for 145 museum specimens (Tables S4 and S5) across geography using morphological indices (Anderson & Daugherty, 1974; Brelsford, Milá & Irwin, 2011). A qualitative index was created by summing up the scores of all 10 morphological characters summarized in Table S6, in which individuals with the *beryllina* phenotype scored 24 on the colour index, and those with the *saucerottei* phenotype scored 0. We plotted index scores as well as single colour characters (rufous patch on the secondary feathers, rufous patch on the primary feathers, belly coloration, tail coloration) against longitude coordinates to examine how morphology varies with geographical distribution.

### Environmental data, palaeodistribution and niche divergence

A complementary species distribution modelling approach was used to frame the information derived from the genetic and morphological analyses within an explicit palaeoecological context. We constructed a species distribution model (SDM; Elith et al., 2011) to predict where the populations of *beryllina*, *cyanura* and *saucerottei* resided during the Last Glacial Maximum (LGM, 21–18,000 years ago) and the Last Interglacial (LIG, 140,000–120,000 years ago). Georeferenced records from museum specimens and from our own collection efforts were used to construct ecological niche models (ENM) based on current climate data using the maximum entropy algorithm implemented in MAXENT v. 3.3.3k (Phillips, Anderson & Schapire, 2006). Data from museum specimens with a precise indication of the collection locality were obtained through http://vertnet.org and the Global Biodiversity Information Facility (GBIF; http://data.gbif.org/species/browse/taxon). Localities were distributed in three groups that correspond to the three genetic groups obtained in the microsatellite analyses. We obtained a total of 158 different records for *beryllina*, 98 for *cyanura* and hummingbirds with intermediate phenotypes, and 32 for *saucerottei*. Present climate layers were drawn from WorldClim (Hijmans et al., 2005; http://www.worldclim.org), and MAXENT was set to randomly use 75% of the values for training and 25% of the values for testing the model. After extracting climatic data for each location from 19 bioclimate layers (c. 1 km^2^ resolution), we tested for correlation among these variables for each of the three groups and chose uncorrelated variables (< 0.7 Pearson correlation coefficient) for further analysis. For each pair of correlated variables, we selected the variable that was most temporally inclusive (Arteaga et al., 2011). Six variables were selected for *beryllina* (BIO1 = annual average temperature, BIO4 = temperature seasonality, BIO7 = annual temperature range, BIO12 = annual precipitation, BIO17 = precipitation in driest quarter, and BIO18 = precipitation in warmest quarter), seven for *cyanura* (BIO1, BIO4, BIO7, BIO12, BIO17, BIO18, BIO19 = precipitation in coldest quarter) and eight for* saucerottei* (BIO1, BIO3 = isothermality, BIO4, BIO7, BIO12, BIO17, BIO18, and BIO19).

We constructed an ENM model for each genetic group using the default convergence threshold (10^−5^) and 500 iterations (Pearson et al., 2007). To assess model performance, we used the area under the receiver operating characteristic curve (AUC; Mertz, 1978). Past climate layers were also drawn from WorldClim for two LGM past climate scenarios developed by the Paleoclimate Modelling Intercomparison Project Phase II (Braconnot et al., 2007): the Community Climate System Model (CCSM; Collins et al., 2004) and the Model for Interdisciplinary Research on Climate (MIROC; Hasumi & Emori, 2004), and the LIG (Otto-Bliesner et al., 2006). Both the CCSM and MIROC climate models simulate LGM climate conditions, with a stronger temperature decrease assumed in CCSM than in MIROC (Otto-Bliesner et al., 2007).

We employed the multivariate method introduced by McCormack, Zellemer & Knowles (2010) to test for divergence or niche conservatism (i.e., closely related species evolve in allopatry under similar ecological conditions; Wiens, 2004; Wiens et al., 2010). The groups show niche conservatism when the difference in the niche occupied by each group is less than the difference between the respective backgrounds. Alternatively, there is niche divergence when the difference in the niche occupied by each group is greater than the difference between the respective backgrounds. The null hypothesis of niche divergence is not rejected when these distances between niches and backgrounds are not different. We tested for niche divergence using climatic data extracted from occurrence points and the eight bioclimate layers used to generate the ENMs, and by drawing minimum convex polygons around the occurrence points of each genetic group (McCormack, Zellemer & Knowles, 2010) using the Hawth’s Tools package. We defined the background characteristics of each group using 1000 random points inside each polygon, and then conducted a principal components analysis (PCA) on these data. The first four axes included a high percentage of the data variance (93.2%) and thus the four components were used in further analyses. We evaluated niche divergence or conservatism on each axis by assessing significance with a null distribution of background divergence, which we created by recalculating the background divergence score over 1000 jackknife replicates with 75% replacement. Analyses were conducted in R packages *vegan* (Oksanen et al., 2012) and *bootstrap* (R Core Team, 2013). We assessed correlation between the four axes and latitude/longitude to have a measure of spatial autocorrelation (McCormack, Zellemer & Knowles, 2010). Using a non-parametric analysis of variance (Kruskal-Wallis) and Dunn’s multiple comparisons test, we also assessed if there is difference in the elevation at which each genetic group is distributed.

## Results

### Genetic diversity and species clustering

High levels of genetic diversity were observed, with the lowest number of haplotypes being 11 in *saucerottei* and the highest, 32 in *cyanura* (Table S7). Haplotype diversity (*h*) and nucleotide diversity (π) values were high, ranging from 0.87 in *saucerottei* to 0.94 in *beryllina* and from 0.005 in *beryllina* to 0.007 in *saucerottei*, respectively (Table S7).

The BI gene tree of mtDNA revealed that *beryllina*, *cyanura*, and *saucerottei*, as well as hummingbirds with intermediate phenotype between *beryllina* and *cyanura*, form a monophyletic group, with genealogical relationships within the ingroup unresolved (i.e., lack of species monophyly; Fig. S1). In addition, *saucerottei* hummingbirds from Central America do not form a monophyletic group with their proposed conspecifics from South America, and some *beryllina* individuals mainly from Jalisco and Michoacán, formed a group separate from the other *beryllina* hummingbirds (Fig. S1). Based on these results and those of McGuire et al. (2014), samples of *saucerottei* from South America were not included in further analyses.

The haplotype network was more informative about the relationships among haplotypes than the BI gene tree was (Figs. 1B–1C). The aligned mtDNA data set (1100 bp) yielded 57 haplotypes. Geographic structuring is observed in the network, however, shared haplotypes between *cyanura* and *beryllina* (H1, H27) were observed at their range edges, from localities close to the Isthmus of Tehuantepec, including Cerro Baúl (Oaxaca, locality 11), Nueva Colombia (Chiapas, locality 16) and Huehuetenango (Guatemala, locality 20). Likewise, *cyanura* hummingbirds that shared haplotypes with *saucerottei* (H48–H51) come from Jinotega (Nicaragua, locality 29), near the Nicaraguan Depression.

In the region located between the Isthmus of Tehuantepec and the Nicaraguan Depression (area known as Nuclear Central America, NCA), hummingbirds with different phenotypes shared the same haplotypes. For instance, those with the *beryllina* phenotype had the same haplotypes as hummingbirds with the *cyanura* or an intermediate phenotype between these two species. Again, the *beryllina* from Jalisco and Michoacán formed a haplogroup (H35–H40) that was more closely connected to *cyanura* and *saucerottei* haplotypes than to other *beryllina* haplotypes (H1–H14; Fig. 1C), and one haplotype (H34) of *saucerottei* from Costa Rica was more closely connected to *cyanura* and *beryllina* haplotypes than to *saucerottei* haplotypes south of the Nicaraguan Depression. Overall, the geographical analysis of the haplotype network suggests a shallow pattern of differentiation across the complete range with mitochondrial haplotype sharing among the three taxa at their distribution margins and certain degree of geographic discordance.

Of the microsatellite loci analysed, there were no linked loci and only three out of a total of 211 comparisons departed from H-W equilibrium after sequential Bonferroni corrections (adjusted *P*-value = 0.00024) at loci Cacu4-8 and HumB1 from Suchitepequez (Guatemala) and HumB1 from Jinotega (Nicaragua). Three genetic groups were detected by STRUCTURE (Figs. 1D–1E), with distributions concordant with known geographic barriers: (i) *beryllina* from northern Mexico to the Isthmus of Tehuantepec, including those from Jalisco and Michoacán, (ii)* cyanura* from the NCA region, and (iii) *saucerottei* from the south of the Nicaraguan Depression. Using STRUCTURE, the estimated logarithm of probability of the data, *Ln* P(*K*), increased linearly from *K* = 1 to *K* = 3 and then plateaued with increased variability (Fig. S2A). The highest Δ*K* also occurred at *K* = 3with large absolute values (Fig. S2B). Runs at *K* = 2 resulted in four different assignment patterns, with *saucerottei* grouping with *beryllina* in seven of the 10 replicate runs and in the remaining three runs with *cyanura* (Fig. S2C).

At *K* = 3, some individuals show signs of admixture (Fig. 1E). Populations of the three groups at the edges of their distributions have hummingbirds with genotypes either from one or the other species at their distribution edges. For example, one population (locality 11) from the eastern side of the Isthmus of Tehuantepec (Cerro Baúl, Oaxaca) has individuals grouped with both *beryllina* (2 out of 6) and *cyanura* (4 out of 6). Similarly, one population (locality 29) from the northern side of the Nicaraguan Depression (Jinotega, Nicaragua) has individuals grouped with both *cyanura* (5 out of 12) and *saucerottei* (4 out of 12), as well as individuals with signs of admixture among the three groups (3 out of 12) (Figs. 1E and S2C). However, *cyanura* from its distribution edges are not more strongly admixed than other *cyanura* (Figs. 1D–1E). Interestingly, some hummingbirds that exhibit evidence of genetic admixture appear scattered throughout the STRUCTURE plot at *K* = 3. Using the model with prior population information, three individuals of *cyanura* were assigned as putative migrants, one from Cerro Baúl (Oaxaca), one from Nueva Colombia (Chiapas) and one from Jinotega (Nicaragua), and one *cyanura* from Jinotega (Nicaragua) was assigned with migrant ancestry.

Congruent with the Bayesian assignment analysis, the principal component analysis of the microsatellite data (Figs. S2C and S2D) showed the same three-species grouping, hereafter *beryllina* (west of the Isthmus of Tehuantepec), *cyanura* (NCA), and *saucerottei* (southern side of the Nicaraguan Depression).

### Demographic history, divergence time and gene flow

Neutrality test values for *beryllina*, *cyanura* and *saucerottei* were not significant using the combined mtDNA data set (Table S7), potentially indicating that their mtDNA is not under selection to maintain ancestral mtDNA or selection for local climatic conditions that are associated with elevation. In the mismatch distribution, sudden demographic expansion (SSD and Hri values) was rejected, indicating that *beryllina*, *cyanura* and *saucerottei* populations have not experienced recent rapid population expansions (Table S7).

The BEAST and IMa analyses of mtDNA sequences revealed recent divergence among species and asymmetrical isolation-with-migration. The time since the most recent common ancestor (TMRCA) for the *beryllina*-*cyanura*-*saucerottei* in-group inferred by the phylogenetic analysis was estimated to be c. 700,000 years before present (0.71 Ma, 95% highest posterior density intervals, 95% HPD, 1.073–0.403 Ma) when using high substitution rates (Lerner et al., 2011) and 2.6 Ma (95% HPD, 3.88–1.41 Ma) when using lower substitution rates (Pacheco et al., 2011). The more recent divergence times obtained using the substitution rates estimated by Lerner et al. (2011) previously used to estimate divergence times in hummingbirds (McGuire et al., 2014; Ornelas et al., 2014) are more appropriate for the taxonomic level of these *Amazilia* species (Hosner, Nyári & Moyle, 2013; Voelker, Bowie & Klicka, 2013).

**Figure 2:**
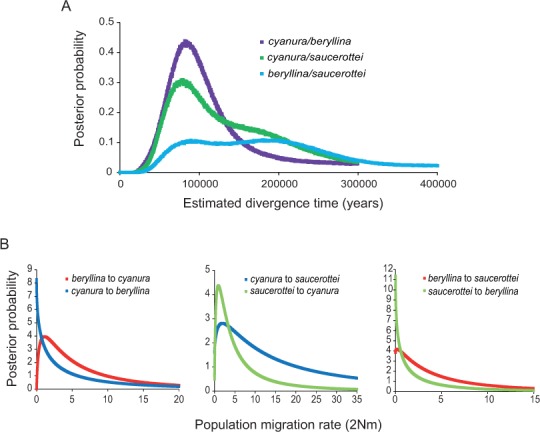
Marginal posterior probability densities for divergence times and migration rates among *Amazilia beryllina*, *A. cyanura*, and *A. saucerottei* using a coalescent approach in IMa and mtDNA. (A) Estimated divergence times between *beryllina*, *cyanura* and *saucerottei*. Estimated divergence dates for the *cyanura/beryllina* and *cyanura/saucerottei* splits are more recent than the date estimated for the *beryllina/saucerottei* split. (B) Estimated gene flow among *beryllina*, *cyanura*, and *saucerottei*. Gene flow was estimated as the population migration rate (2Nm), which is equivalent to the historical average number of immigrants between species per generation.

The divergence times inferred using IMa indicate that the time since divergence between *beryllina* and *saucerottei* occurred c. 300,000 years before present (0.34 Ma, Table 1), with a wide range and flat distribution of posterior probabilities for *t* (Fig. 2A); *cyanura* and *saucerottei* and *cyanura* and *beryllina* diverged more recently (Table 1), within the last 100,000 years before present, with clearly defined unimodal peaks of posterior probabilities for *t* around 0.13 and 0.11 Ma, respectively (Fig. 2A), when using the substitution rates estimated by Lerner et al. (2011). Asymmetrical gene flow occurred during the process of speciation in a north-to-south direction, with higher migration values from *beryllina* to *cyanura* and *saucerottei* than in the opposite direction; gene flow between *cyanura* and *saucerottei* was symmetrical during divergence (Table 1 and Fig. 2B).

**Table 1:** Results of isolation-with-migration model for the splits among *Amazilia beryllina*, *A. cyanura* and *A. saucerottei*.

	Substitution rates according to Lerner et al. (2011)								
	Survival rate 0.30			Survival rate 0.52					
	N1	N2	Na	N1	N2	Na	t	N*m*1	N*m*2
*A. beryllina* vs. *A. cyanura*									
Mean	96	242	95	76	191	75	113	1.459	6.492
HPD95Lo	45	150	12	35	118	10	6	0.019	0.740
HPD95Hi	180	373	275	142	293	217	269	9.422	26.342
*A. saucerottei* vs. *A. cyanura*									
Mean	195	215	116	153	170	91	134	5.755	5.195
HPD95Lo	89	134	11	70	106	9	47	0.176	0.493
HPD95Hi	387	327	294	305	257	231	276	27.662	21.667
*A. beryllina* vs. *A. saucerottei*									
Mean	83	220	101	65	173	80	344	0.992	4.538
HPD95Lo	38	102	5	30	80	4	59	0.011	0.086
HPD95Hi	154	431	212	122	339	167	896	7.016	28.437

	Substitution rates according to Pacheco et al. (2011)								
	Survival rate 0.30			Survival rate 0.52					
	N1	N2	Na	N1	N2	Na	t	N*m*1	N*m*2
*A. beryllina* vs. *A. cyanura*									
Mean	409	1,033	405	322	814	319	483	1.459	6.492
HPD95Lo	190	639	53	150	503	42	27	0.019	0.740
HPD95Hi	766	1,591	1,174	603	1,253	924	1,146	9.422	26.342
*A. saucerottei* vs. *A. cyanura*									
Mean	830	919	493	654	724	388	570	5.755	5.195
HPD95Lo	378	572	47	298	451	37	198	0.176	0.493
HPD95Hi	1,650	1,393	1,252	1,300	1,097	986	1,176	27.662	21.667
*A. beryllina* vs. *A. saucerottei*									
Mean	353	936	431	278	737	340	1,469	0.992	4.538
HPD95Lo	162	435	22	128	342	17	253	0.011	0.086
HPD95Hi	659	1,836	905	519	1,446	713	3,818	7.016	28.437

Final runs of BAYESASS yielded consistently low estimates of contemporary gene flow (<1%) between the genetic groups of *Amazilia* within the last few generations and all estimates of migration (*m*) had 95% confidence intervals that approached zero, indicating little to no recent migration between genetic groups (Table 2). Estimates of historical migration rates (*M*) calculated using MIGRATE revealed asymmetrical migration among groups over the long term, with estimates of *M* ranging from 2 to 13 and in general decreasing from north to south (Table 2). The number of migrants per generation (*N_e_m*) ranged from 0.58 to 2.18. Results from the Mantel test were not significant (*r* = −0.40, *P* = 0.66) indicating that the two matrices of contemporary and historical migration values are not correlated with each other, which implies that the rate and intensity of migration between groups decreased from past to present.

**Table 2:** BAYESASS and MIGRATE estimates of contemporary and historical migration rates (95% confidence intervals), respectively, based on microsatellites data between groups of *Amazilia* hummingbirds. The recipient (sink) populations are shown in the left side, and the source (donor) populations are across the top.

	Source population		
Recipient population	*A. beryllina*	*A. cyanura*	*A. saucerottei*
BAYESASS			
Contemporary migration rates (*m*)		chi	
*A. beryllina*	0.98 (0.95–1.00)	0.01 (0.00–0.04)	0.01 (0.00–0.02)
*A. cyanura*	0.02 (0.00–0.03)	0.97 (0.95–1.00)	0.01 (0.00–0.02)
*A. saucerottei*	0.07 (0.01–0.13)	0.03 (0.00–0.07)	0.90 (0.83–0.97)
MIGRATE			
Historical migration rates (*M*)			
*A. beryllina*	–	10.66 (7.27–16.20)	2.92 (0.27–5.40)
*A. cyanura*	4.47 (1.40–7.47)	–	1.96 (0.00–3.87)
*A. saucerottei*	3.37 (0.40–6.33)	12.61 (5.73–21.20)	–
Number of migrants per generation (*N_e_m*)			
*A. beryllina*	–	1.44 (0.00–8.64)	0.39 (0.00–2.88)
*A. cyanura*	1.42 (0.00–5.35)	–	0.62 (0.00–2.77)
*A. saucerottei*	0.58 (0.00–3.39)	2.18 (0.00–12.37)	–

### Morphological variation

**Figure 3:**
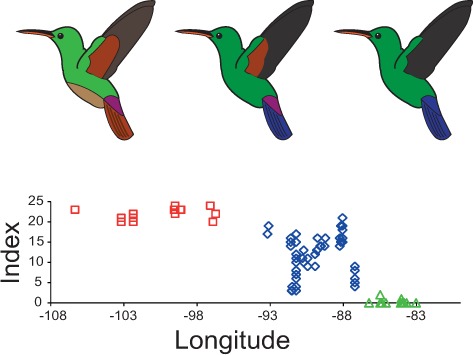
Plumage colouration patterns of *A. beryllina*, *A. cyanura* and *A. saucerottei*. Hummingbird drawings indicate species-specific plumage characteristics, mainly the presence of a rufous patch on the wing and tail feather colouration. Genotyped individuals were scored using a qualitative index that summed up the scores for 10 plumage colouration characters.

The plumage colouration analyses indicate that *beryllina* and *saucerottei* at the north/southern limits of the combined range of the species complex are morphologically homogenous. In contrast, *cyanura* samples in the central portion of the range (NCA) are the morphologically intermediate of the other two species, with different phenotypes occurring at the same locality according to the plumage colouration index (Figs. 3 and S3A). Although *cyanura* hummingbirds have intermediate phenotypes, some of their characteristics are more similar to those of either *beryllina* or *saucerottei* (Fig. S3). For example, the amount of rufous in the wing patch of secondary feathers of some *cyanura* is more similar to that of *beryllina* (Fig. S3B), and the lack of rufous on the wing patch in primary feathers of some *cyanura* is similar to that of *saucerottei* (Fig. S3C). Belly colouration in *cyanura* can be *beryllina* golden-green or *saucerottei* emerald-green (Fig. S3D). Tail colouration is one of the more variable characters in *cyanura* (Fig. S3E), where *cyanura* samples can be *beryllina* rufous-brown, *saucerottei* metallic blue, a mixture of these colours, or completely purple in some hummingbirds (Fig. S4). Nonetheless, those with different plumage colouration can have the same mitochondrial haplotypes and be part of the same group according to the microsatellite analysis.

### Palaeodistribution and niche divergence

**Figure 4:**
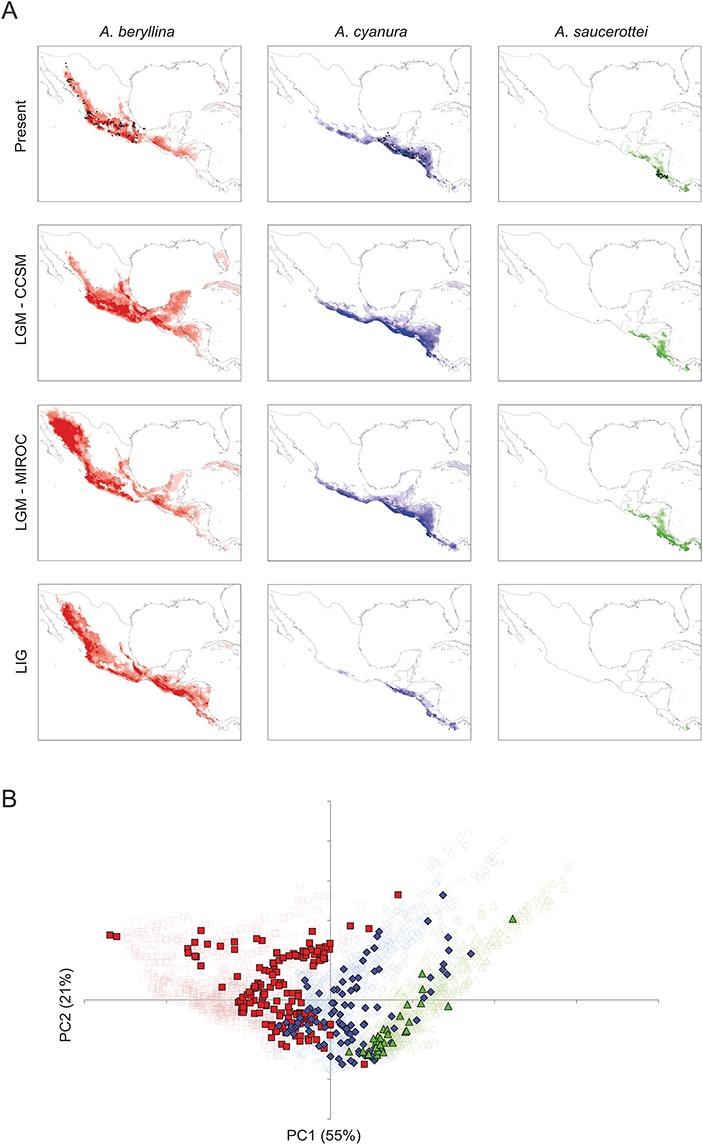
Present and past distribution models and environmental space for *Amazilia beryllina*, *A. cyanura*, and *A. saucerottei*. (A) Species distribution models generated with MaxEnt v. 3.3.3k for *beryllina*, *cyanura* and *saucerottei* for the present, Last Glacial Maximum (LGM, MIROC), Last Glacial Maximum (LGM, CCSM), and Last Interglacial (LIG). Darker shading indicates the most probable predicted distribution. (B) Principal components analysis showing occurrence points (closed symbols) and 1000 random points from the background (open symbols) where each of the three species is found; *beryllina* (red squares), *cyanura* (blue diamonds), and *saucerottei* (green triangles).

The current distribution models (all AUC values >0.90) yielded a good fit for the current geographic distribution of *beryllina* to the west of the Isthmus of Tehuantepec, *saucerottei* to the south of the Nicaraguan Depression, and* cyanura* between these geographical barriers (Fig. 4A). These models suggest that the environmental characteristics for the three species can be found mainly within their current distribution limits, though these geographical environments overlap between species.

The palaeodistribution modeling revealed that suitable habitat for populations of all three species expanded under LGM conditions particularly across the barriers posed by the Isthmus of Tehuantepec and the Nicaraguan Depression, which would have allowed population expansion and secondary contact (Fig. 4A). The predictions of the CCSM and MIROC models were similar for *cyanura* and *saucerottei* whereas for *beryllina* the CCSM and MIROC models differed, with the expansion of suitable habitat in the southern, central and northwest portions of Mexico according to the projections of the CCSM and MIROC models, respectively. Interestingly, predictions under LIG conditions revealed that areas of suitable habitat for *beryllina* populations extended southward compared to predicted distributions under current or LGM conditions to include those of *cyanura* and *saucerottei*. In contrast, the predicted areas of suitable habitat for *cyanura* and *saucerottei* contracted during the LIG, with conditions of suitable habitat for *saucerottei* almost disappearing from the region (Fig. 4A).

Tests of niche conservatism and divergence on four PC axes showed evidence for niche conservatism on niche axis 1 (55% of variation; Table 3) associated with annual precipitation and temperature seasonality. In contrast, evidence for niche divergence was detected on niche axes 2 and 3 (21% and 10% of total variation, respectively) associated with precipitation seasonality (Table 3). Niche conservatism on niche axis 1 is evident from the overlap of species occurrences along PC1 when compared with PC2 (Fig. 4B). Spatial autocorrelation cannot be ruled out as the driving force behind this pattern because niche axis 1 was highly correlated with latitude and longitude (Table 3). However, elevation ranges differ among species (mean ± SD; *beryllina*, 1375 ± 700 m above sea level; *cyanura*, 921 ± 526 m a.s.l.; *saucerottei*, 542 ± 585 m a.s.l.), where *beryllina* inhabits higher elevations and *saucerottei*, lower elevations (Kruskal-Wallis = 51.226, *P* < 0.0001; Dunn’s multiple comparisons test, *P* < 0.05).

**Table 3:** Divergence on niche axes among *Amazilia beryllina*, *A. cyanura* and *A. saucerottei*. **Bold** values indicate significant niche divergence (D) or conservatism (C) compared with a null distribution (in parentheses) based on background divergence among their respective geographical ranges.

Paired comparison	Niche axes			
	PC1	PC2	PC3	PC4
*A. beryllina* vs. *A. cyanura*	**0.88 C** (1.25, 1.44)	**0.59 D** (0.05, 0.33)	0.30 (0.16, 0.53)	**0.20 C** (0.60, 0.91)
*A. beryllina* vs. *A. saucerottei*	**1.36 C** (2.03, 2.22)	**1.07 D** (0.40, 0.73)	**0.38 D** (0.00, 0.21)	**0.89 D** (0.11, 0.46)
*A. cyanura* vs. *A. saucerottei*	**0.47 C** (0.70, 0.87)	0.49 (0.24, 0.59)	**0.68 D** (0.21, 0.60)	1.10 (0.93, 0.17)
% explained variance	54.9	20.7	9.9	7.7
Variables with high values	BIO3	BIO17	BIO1	BIO18
	BIO4	BIO18		
	BIO7			
	BIO12			
	BIO19			
Biological interpretation	Temperature seasonality and annual precipitation	Precipitation seasonality	Temperature	Precipitation in warm season
Latitude correlation	−0.82	0.60	−0.27	0.21
Longitude correlation	0.79	−0.50	0.16	−0.25

## Discussion

Two general patterns of genetic divergence were found among *beryllina*, *cyanura* and *saucerottei* hummingbirds with overlapping distributions in Mesoamerica. The mtDNA analyses revealed a lack of species monophyly, recent divergence among species, and asymmetrical isolation-with-migration, whereas the microsatellite analyses provide evidence for three genetic clusters with distributions corresponding to isolation caused by the Isthmus of Tehuantepec and the Nicaraguan Depression, with some hummingbirds showing signs of admixture. On the other hand, samples from the central portion of the range, *cyanura* according to microsatellites, have phenotypes that are intermediate to those from the northern (*beryllina*) and southern (*saucerottei*) limits of the combined range. Despite the potential for spatial autocorrelation, the distribution models give evidence of niche conservatism and potential range expansions and contractions promoted by Pleistocene climatic oscillations that may have contributed to historical isolation and secondary contact. These results indicate that the three genetic clusters are very young and their evolutionary history has occurred in the presence of gene flow, despite the difficulty in distinguishing the processes of incomplete lineage sorting from complete introgression using the currently available data.

### Genetic and morphological patterns

The phylogeographic pattern of mtDNA haplotypes and microsatellites were discordant, with haplotype sharing of mtDNA between clades showing no discernible geographic pattern in the distribution of non-monophyletic mtDNA species clades, and microsatellite analyses showing a pattern of differentiation between three groups isolated by topographic barriers, the Isthmus of Tehuantepec and the Nicaraguan Depression with some admixed individuals. These particular geographic lowlands recurrently have imposed impassable or nearly impassable barriers to gene flow for several montane plant (e.g., Gutiérrez-Rodríguez, Ornelas & Rodríguez-Gómez, 2011; Ornelas & González, 2014; Ornelas & Rodríguez-Gómez, 2015) and bird species (e.g., González, Ornelas & Gutiérrez-Rodríguez, 2011; Rodríguez-Gómez, Gutiérrez-Rodríguez & Ornelas, 2013; Rodríguez-Gómez & Ornelas, 2014, 2015; Ortiz-Ramírez et al., 2016). However, the haplotype network yielded two groups with unexpected haplotype relationships, with haplotypes from the western side of the Isthmus of Tehuantepec were closely connected to some NCA haplotypes, whereas some other NCA haplotypes were linked to haplotypes from the south of the Nicaraguan Depression. This mtDNA network pattern supports incomplete lineage sorting or genetic introgression, as suggested by the Bayesian inference analysis. The discordant patterns evident between the mtDNA haplotype network and nuclear microsatellites in delimiting three genetic groups is likely the result of their different inheritance patterns and mutation rates and, therefore, evidence of recent isolation (i.e., incomplete mtDNA lineage sorting) and secondary contact in the recent past (hybridization and partial introgression; Toews & Brelsford, 2012). Therefore, the geographical barriers suggested by microsatellite loci might have some kind of permeability for this hummingbird species complex, since the hummingbirds collected in the surrounding areas exhibit genetic admixture, some of them being assigned as putative hybrids or as having migrant ancestry.

The haplotype network and BI tree showed that some *beryllina* mainly from Jalisco and Michoacán, are more closely related to *cyanura* and *saucerottei* than to other *beryllina*, and one *saucerottei* from Costa Rica was more closely connected to *cyanura* and *beryllina* than to *saucerottei* south of the Nicaraguan Depression (i.e., incomplete lineage sorting). However, these same hummingbirds were assigned to the *beryllina* or *saucerottei* clusters in the microsatellite analyses, suggesting that species have recently stopped exchanging genes (mtDNA haplotype sharing and admixed individuals mainly observed at the contact zones) yet still possess haplotypes very closely related to those of geographically distant populations (Funk & Omland, 2003). Whether discordance in genetic patterns in mtDNA and microsatellites is explained by selection that may favour some mitochondrial variants over others due to selection to maintain ancestral mtDNA or selection for environmental gradients (Cheviron & Brumfield, 2009), allowing populations to remain as a different mitochondrial group is unlikely according to the neutrality test results. We must be careful, however, about over-interpreting these results until additional genome-wide markers become available because incomplete lineage sorting and introgressive hybridization can produce similar genetic signatures (i.e., haplotype sharing between species; Rheindt & Edwards, 2011), particularly during species formation (Qu et al., 2012; Wang et al., 2014; Rodríguez-Gómez & Ornelas, 2015).

The divergence times of the *Amazilia* species at the Isthmus of Tehuantepec and Nicaraguan Depression during the Pleistocene, however, are consistent with the hypothesis that populations diverged when species’ ranges decreased because of forest contraction and fragmentation during the interglacial cycles with warmer climates, and that during the glacial cycles secondary contact allowed migration because of forest expansion to lowland barriers, which influenced the colonization and directionality of gene flow and introgressive hybridization among populations of these *Amazilia* species.

Scoring genotyped hummingbirds by plumage colouration characters showed that *beryllina* and *saucerottei* are phenotypically homogeneous across their ranges. In contrast, plumage colour analyses revealed that samples in the central portion of the range, *cyanura*, have phenotypes that are intermediate to those at the northern and southern limits of the combined range, *beryllina* and *saucerottei*. This is not surprising given that intermediate phenotypes between *beryllina* and *cyanura* were first reported about 100 years ago by Ridgway (1911), and others have since proposed interspecific hybridization in Guatemalan *Amazilia* hummingbirds as the most plausible and common explanation for the remarkable colour plumage variation found in this region (Howell & Webb, 1995, Schuchmann, 1999). However, interspecific hybridization cannot be comprehended without a more thorough geographic sampling. The phylogeographic structure observed in microsatellite data and implying the existence of three genetic clusters does not reflect the clinal variation of plumage colouration. It is possible that the observed variation in plumage colour within the central range of the species complex represents phenotypic responses to local environmental conditions, where these colour changes rapidly occurred regardless of the historical fragmentation of populations by the Isthmus of Tehuantepec and Nicaraguan Depression. Although *cyanura* is almost genetically isolated from the other two groups, the lack of a distinct phenotype within its range suggests that a single colour pattern has not become fixed in this species, and that variation in plumage colouration is maintained as a balanced polymorphism, or that mating in *cyanura* is no assortative with respect to plumage between barriers (see also Magalhaes et al., 2015). We hypothesize that the observed phenotypic diversity of *cyanura* is a result of the diversification of a single ancestral population, in which the greater phenotypic and genetic diversity of *cyanura* was maintained by the high migration rates of *beryllina* and *saucerottei* to the *cyanura* range during secondary contact, and that the reduced phenotypic variation in *beryllina* and *saucerottei* is the result of founder effects across the isthmus and depression barriers. Additional genome-wide data and fine-scale sampling is needed to distinguish between these alternatives, explain the wide phenotypic diversity and definitively assess the degree of introgression, if any, between taxa.

### Genetic introgression mediated by Quaternary climatic oscillations

The TMRCA for the entire *beryllina*/*cyanura*/*saucerottei* in-group was estimated to be c. 700,000 years before present, which coincides with the start of intense glacial cycles (Webb & Bartlein, 1992). This divergence time has been reported for species/subspecies mtDNA level splits in other Mesoamerican avian groups (e.g., Barrera-Guzmán et al., 2012; Ornelas et al., 2013; Malpica & Ornelas, 2014; Rodríguez-Gómez & Ornelas, 2014), suggesting an important role of Pleistocene glacial cycles in autochthonous diversification. Given that divergence time estimates were obtained with mtDNA only (not likely to resemble the species phylogeny), divergence times might differ substantially if estimated with both mtDNA and nuDNA sequence data (McCormack, Zellemer & Knowles, 2010).

The IMa analysis based on mtDNA showed that the time since the divergence of *beryllina* and *saucerottei* was c. 300,000 years ago. However, posterior distributions of the divergence time parameter had a peak at c. 120,000 years. The curve then plateaued to the right and then increased to a second similar peak at c. 200,000 years, preventing the lower and upper bounds from being estimated accurately (Hey & Nielsen, 2004, 2007). Time since divergence of *cyanura* from the other two species was estimated in both cases as c. 120,000 years ago. These analyses produced posterior distributions of divergence time parameters with clear peaks and bounds within the prior distribution, suggesting that these estimates are more reliable, and overlap substantially with the first peak of the posterior distributions of divergence time between *beryllina* and *saucerottei*. Despite caveats about divergence time estimation and assuming selective neutrality according to neutrality tests, these divergence times suggest an allopatric phase between *cyanura* and *beryllina* populations north of the Isthmus of Tehuantepec and between *cyanura* and *saucerottei* populations south of the Nicaraguan Depression during the interglacials.

Divergence between *cyanura* and the other two species at the Isthmus of Tehuantepec and Nicaraguan Depression coincided with the LIG, when suitable habitat for *beryllina* extended to cover most of Central America according to the palaeodistribution modelling. When its range was extended southward, *beryllina* could have come into contact with *cyanura* and *saucerottei*, inhabiting more restricted areas of suitable habitat, thus producing hybrids and leaving a genetic signal of mtDNA introgression across the entire range of the species complex. Secondary contact might have occurred as well during the LGM across the Isthmus of Tehuantepec and Nicaraguan Depression owing to the expansion of mountain vegetation into the lowlands (Ramírez-Barahona & Eguiarte, 2013), which enabled mtDNA gene flow between previously diverged species between the LIG and LGM. In agreement with this, our IMa analysis suggested that the mtDNA gene flow was asymmetrical, with high gene flow from *beryllina* to *cyanura* and *saucerottei*, and negligible gene flow in the reverse direction. Furthermore, species such as *beryllina*, distributed at higher elevations, may be more prone to invading the lowlands than species distributed at lower elevations such as *cyanura* and *saucerottei* would be to colonize the highlands because of the physiological limits imposed by elevation (Altshuler & Dudley, 2002). This corresponds to the almost symmetrical mtDNA gene flow between *cyanura* and *saucerottei* across the Nicaraguan Depression.

The distribution models give evidence of niche conservatism and potential range expansions and contractions that may have contributed to historical isolation and secondary contact. Tests of niche conservatism and divergence along the niche axis 1 (55% of variation) suggest that these closely related species have retained their ecological niche characteristics over evolutionary time (niche conservatism; Wiens et al., 2010). However, niche divergence was suggested on niche axes 2 and 3 (30% of variation), where the occurrence points of the three species do not overlap completely in ecological space. The observed ecological divergence is coupled with nuclear genetic breaks at the Isthmus of Tehuantepec and Nicaraguan Depression, which separates *cyanura* from *beryllina* hummingbirds north of the isthmus and from *saucerottei* south of the depression barrier. Thus, despite sharing some environmental characteristics, the species niches are not totally conserved and there is some differentiation among species in environmental space, primarily associated with differences in precipitation seasonality and elevation ranges (McCormack, Zellemer & Knowles, 2010).

### Contemporary and historical migration rates

Our analysis showed that historical levels of migration between genetically distinct groups of *Amazilia* (*beryllina*, *cyanura*, *saucerottei*) were high and different in magnitude than contemporary levels of migration. High levels of historical migration rates are not surprising because extant populations might have experienced secondary contact and range contact during the glacial periods of the Pleistocene, as suggested by species distribution modelling, making dispersal across the isthmus and depression barriers likely. More surprising is that contemporary migration rates are low because assignment analyses identified genetic admixture both at the contact zones and scattered throughout the sampled ranges. These results strongly imply that the high levels of structure currently observed are a consequence of the limited dispersal of these hummingbirds across the isthmus and depression barriers, and that the admixed individuals scattered throughout the STRUCTURE plot were likely pure, incorrectly assigned individuals at these levels of differentiation (Abbott et al., 2013; Putman & Carbone, 2014).

From the biogeographical perspective of introgression, our study provides the first genetic and morphological evidence that these species of *Amazilia* constitute an avian species complex with a clinal plumage colouration pattern in the Mesoamerican region. The three hummingbird species analysed responded to Quaternary climatic oscillations with expansion-contraction cycles in their geographic ranges. These changes in distribution were likely involved in producing periods of isolation and allopatric speciation, followed by secondary contact and genetic introgression. It is of interest that other sets of closely related hummingbird species and other highland bird species offer an opportunity to understand implications of past climate change in the same geographical area, as these species also have a very complex colouration pattern and share the same geographic barriers in their distribution, from northern Mexico to northern South America. This suggests that the processes that brought intermediate phenotypes in this *Amazilia* species complex need to be further studied because the implications of genetic introgression in the diversification of this avian group are not understood.

### Taxonomic implications

Our results contribute two major findings that have taxonomic implications. First, they confirm that Central American *A. saucerottei hoffmanni* and *A. saucerottei* subspecies from South America (*warscewiczi*, *saucerottei*, *braccata*) are two different taxa with considerable genetic divergence. Thus, our results and those of McGuire et al. (2014) suggest that they belong to different groups of *Amazilia* species, and support the proposal of Stiles & Skutch (1989) that the Central American *A. saucerottei hoffmanni* is a different species (*A. sophiae*) based on notorious behavioural and vocal differences. Further multilocus analysis of samples from Colombia and Venezuela is necessary to assess divergence patterns between South American populations of *saucerottei* and other *Amazilia* species. Second, our results reveal three genetic clusters that have distributions consistent with known geographic barriers, with some hummingbirds showing signs of admixture and intermediate phenotypes in the central portion of the range: (1) *A. beryllina* located west of the Isthmus of Tehuantepec, (2) *A. cyanura* between the Isthmus of Tehuantepec and the Nicaraguan Depression (Nuclear Central America), and (3) *A. saucerottei hoffmanni* syn. *A sophiae* located between the Nicaraguan Depression and the Isthmus of Panama. Increased genomic resources and behavioural data will likely confirm that they constitute separate taxonomic species. These findings are in line with several examples of recent speciation events reported in hummingbird species, some of which show congruent genetic and phenotypic divergence (González, Ornelas & Gutiérrez-Rodríguez, 2011; Rodríguez-Gómez & Ornelas, 2015), while others show genetic divergence and no apparent plumage divergence (Rodríguez-Gómez, Gutiérrez-Rodríguez & Ornelas, 2013; Licona-Vera & Ornelas, 2014; Malpica & Ornelas, 2014). Additional genome-wide markers might help to elucidate the evolutionary history of this young system, in which detecting divergence in a few genes under selection may be critical for proper species delimitation.

## Supplemental Information

10.7717/peerj.1556/supp-1 Supplemental Information 1 Collecting localities for *Amazilia beryllina*, *A. cyanura*, *A. saucerottei* samples and those for individuals with intermediate phenotypes examined genetically.

10.7717/peerj.1556/supp-2 Supplemental Information 2 Species names, voucher information, localities of origin, and GenBank accession numbers for the specimens examined genetically and the outgroup taxa used in this study.

10.7717/peerj.1556/supp-3 Supplemental Information 3 Frequency and number of base pairs (bp), variable sites, and parsimony informative sites for the gene partitions, and DNA substitution models applied to each partition.

10.7717/peerj.1556/supp-4 Supplemental Information 4 Collection localities for the *Amazilia beryllina*, *A. cyanura*, *A. saucerottei* samples and those for individuals with intermediate phenotypes examined morphologically in this study.

10.7717/peerj.1556/supp-5 Supplemental Information 5 Species names, voucher information, and localities of origin for the specimens examined morphologically in this study.

10.7717/peerj.1556/supp-6 Supplemental Information 6 Colour characters and index scores used to assess plumage colour variation.

10.7717/peerj.1556/supp-7 Supplemental Information 7 Summary statistics, neutrality tests and population expansion tests for *Amazilia beryllina*, *A. cyanura* and *A. saucerottei*. nsion events. Significant (*P* £ 0.05) SSD and Hri values indicate deviations.

10.7717/peerj.1556/supp-8 Supplemental Information 8 Phylogenetic relationships of mitochondrial haplotypes based on Bayesian inference.

10.7717/peerj.1556/supp-9 Supplemental Information 9 Inference of *K*, the most probable number of clusters based on 12 microsatellite loci for 145 hummingbirds using STRUCTURE v. 2.3.4.

10.7717/peerj.1556/supp-10 Supplemental Information 10 Plumage colour variation in *A. beryllina* (red squares), *A. cyanura* (blue diamonds) and *A. saucerottei* (green triangles).

10.7717/peerj.1556/supp-11 Supplemental Information 11 *Amazilia cyanura* intraspecific variation in tail colouration. Morphological differentiation includes rufous (above left) and blue (below right) forms that resemble the *Amazilia beryllina* and *A. saucerottei* forms, respectively. Figure 4: Present and past distribution models and environmental space for *Amazilia beryllina*, *A. cyanura*, and *A. saucerottei*. (A) Species distribution models generated with MaxEnt v. 3.3.3k for *beryllina*, *cyanura* and *saucerottei* for the present, Last Glacial Maximum (LGM, MIROC), Last Glacial Maximum (LGM, CCSM), and Last Interglacial (LIG). Darker shading indicates the most probable predicted distribution. (B) Principal components analysis showing occurrence points (closed symbols) and 1000 random points from the background (open symbols) where each of the three species is found; *beryllina* (red squares), *cyanura* (blue diamonds), and *saucerottei* (green triangles).

## Additional Information and Declaration

### Field Study Permissions

The following information was supplied relating to field study approvals (i.e., approving body and any reference numbers): Our fieldwork was conducted with the permission of the Mexico’s Secretaría de Medio Ambiente y Recursos Naturales, Subsecretaría de Gestión para la Protección Ambiental, Dirección General de Vida Silvestre (permit numbers Dirección de Vida Silvestre, INE, SEMARNAP, D00-02/3269, SGPA/DGVS/07701/11) and the Consejo Nacional de Áreas Protegidas (CONAP) in Guatemala (permit number 14815). We thank the staff and owners of private reserves (San Jerónimo Miramar y Quixayá, Santa Rosa Sumatán, La Igualdad, Los Tarrales, Patrocinio, El Pilar, Cabaña Suiza, Nueva Granada, El Rosario, Irlanda), Municipalidad de Todos Santos Cuchumatán, and the Asociación de Reservas Naturales Privadas de Guatemala for allowing us to access to these areas.

### DNA Deposition

The following information was supplied regarding the deposition of DNA sequences: GenBank Accession numbers KM198972–KM199267.

### Data Deposition

The following information was supplied regarding data availability: The IMa input files, and morphological and microsatellite data are at Dryad: doi:10.5061/dryad.2c088
