# Supplementary material for: Historical and current introgression in a Mesoamerican hummingbird species complex: a biogeographic perspective
Source: PeerJ. 2016 Jan 12;4:e1556. doi: 10.7717/peerj.1556 (PMC4715438; doi:10.7717/peerj.1556)
Supplement: Supplemental Information 1 [file peerj-04-1556-s001.docx]

**Table S1 Collecting localities for *Amazilia beryllina*, *A. cyanura*, *A. saucerottei* samples and those for individuals with intermediate phenotypes examined genetically**. *n* = sample size (numbers in parentheses); MX = Mexico, GT = Guatemala, NIC = Nicaragua, CR = Costa Rica. *Subspecies according to geography and current taxonomy (Dickinson & Remsen 2013: *Amazilia beryllina* (*viola*, *beryllina*, *lichtensteini*, *sumichrasti*, *devillei*), *A. cyanura* (*guatemalae*, *cyanura*, *impatiens*), and *A. saucerottei* (*hoffmanni*, *warscewiczi*, *saucerottei*, *braccata*). **Phenotype, b = *beryllina*, c = *cyanura*, s = *saucerottei*, i = intermediate between *beryllina* and *cyanura*. ***Genetic group according to microsatellites results in the present study.

| **No.** | **Locality** | **Subspecies*** | **Phenotype****  **(*n*)** | **Genetic group***** | **Latitude**  **N** | **Longitude W** | **Elevation**  **(m)** |
| --- | --- | --- | --- | --- | --- | --- | --- |
| 1 | MX: Sinaloa, Choix | *viola* | b (5) | *A. beryllina* | 26.81667° | 108.20000° | 1325 |
| 2 | MX: Veracruz, Xalapa | *beryllina* | b (8) |  | 19.54000° | 96.92750° | 1460 |
| 3 | MX: Jalisco, Nevado de Colima | *viola* | b (2) |  | 19.60000° | 103.61667° | 3000 |
| 4 | MX: Jalisco, San José de Gracia | *viola* | b (2) |  | 20.67411° | 102.57170° | 1900 |
| 5 | MX: Michoacán, Morelia | *viola* | b (3) |  | 19.70595° | 101.19498° | 1900 |
| 6 | MX: DF, Pedregal | *beryllina* | b (8) |  | 19.31587° | 99.19260° | 2320 |
| 7 | MX: Guerrero, Omiltemi | *viola* | b (5) |  | 17.53333° | 99.63333° | 1920 |
| 8 | MX: Oaxaca, San Gabriel Mixtepec | *beryllina* | b (4) |  | 16.09460° | 97.08160° | 700 |
| 9 | MX: Oaxaca, Piedra Larga | *beryllina* | b (1) |  | 16.14444° | 97.06111° | 1000 |
| 10 | MX: Oaxaca, San Baltazar | *beryllina* | b (1) |  | 16.79626° | 96.30372° | 1600 |
| 11 | MX: Oaxaca, Cerro Baúl | *beryllina* | b (6) | *A. cyanura* | 16.51417° | 94.19750° | 1140 |
| 12 | MX: Chiapas, Jitotol | *sumichrasti* | b (1) |  | 17.13806° | 92.87806° | 1640 |
| 13 | MX: Chiapas, Pueblo Nuevo | *sumichrasti* | b (1) |  | 17.14167° | 92.88556° | 1640 |
| 14 | MX: Chiapas, Monte Sinaí | *sumichrasti* | b (1) |  | 16.64306° | 93.76222° | 650 |
| 15 | MX: Chiapas, Salvador Urbina | *lichtensteini* | b (6) |  | 15.76278° | 92.72389° | 910 |
| 16 | MX: Chiapas, Nueva Colombia | *lichtensteini* | b (11) |  | 15.69611° | 92.72111° | 1380 |
| 17 | MX: Chiapas, Escuintla | *guatemalae* | c (1) |  | 15.45671° | 92.51349° | 1500 |
| 18 | MX: Chiapas, Tapachula | *guatemalae* | c (6) |  | 15.17346° | 92.33599° | 1095 |
| 19 | MX: Chiapas, Tacaná | *guatemalae* | c (1) |  | 15.09389° | 92.10028° | 2090 |
| 20 | GT: Huehuetenango, Todos Santos Cuchumatán | *lichtensteini* | b (5) |  | 15.59093° | 91.68322° | 1800 |
| 21 | GT: San Marcos, El Tumbador | *guatemalae* | c (1) |  | 14.84948° | 91.88728° | 1420 |
| 22 | GT: San Marcos, La Reforma | *guatemalae* | c (1), s (1) |  | 14.82732° | 91.82119° | 1490 |
| 23 | GT: Quetzaltenango, El Palmar | *guatemalae* | c (6) |  | 14.66428° | 91.60941° | 750 |
| 24 | GT: Suchitepéquez, Patulul | *devillei, guatemalae* | b (4), c (3), i (17) |  | 14.54584° | 91.14759° | 1135 |
| 25 | GT: Chimaltenango, San Pedro Yepocapa | *devillei* | b (2), i (2) |  | 14.44156° | 91.06678° | 690 |
| 26 | GT: Guatemala, Mixco | *devillei* | b (2) |  | 14.61691° | 90.61850° | 1840 |
| 27 | GT: Sacatepéquez, San Cristóbal El Bajo | *devillei* | b (2) |  | 14.54216° | 90.71423° | 1700 |
| 28 | GT: Chiquimula, Esquipulas | *devillei* | b (3) |  | 14.55575° | 89.34424° | 990 |
| 29 | NIC: Jinotega, El Jaguar | *cyanura, hoffmanni* | c (11), s (1) |  | 13.23347° | 86.05193° | 1280 |
| 30 | NIC: Granada | *hoffmanni* | s (10) | *A. saucerrottei* | 11.83420° | 85.500° | 600 |
| 31 | CR: Guanacaste | *hoffmanni* | s (10) |  | 10.85361° | 85.60889° | 300 |
|  | TOTAL |  | 154 |  |  |  |  |
