# Supplementary material for: Historical and current introgression in a Mesoamerican hummingbird species complex: a biogeographic perspective
Source: PeerJ. 2016 Jan 12;4:e1556. doi: 10.7717/peerj.1556 (PMC4715438; doi:10.7717/peerj.1556)
Supplement: Supplemental Information 2 [file peerj-04-1556-s002.docx]

**Table S2** **Species names, voucher information, localities of origin, and GenBank accession numbers for the specimens examined genetically and the outgroup taxa used in this study**. Museum abbreviations used in voucher numbers: INECOL = Instituto de Ecología, AC, Xalapa, Veracruz, Mexico; UWBM = Burke Museum of Natural History and Culture; MCZ = Museum of Comparative Zoology; MUSHNAT = Museo de Historia Natural, Universidad de San Carlos de Guatemala; LSUMNS = Collection of Genetic Resources, Louisiana State University Museum of Natural Science; CZRAV = Colección Zoológica Regional Aves, Instituto de Historia Natural y Ecología. MX = Mexico, GT = Guatemala, ES = El Salvador, NIC = Nicaragua, CR = Costa Rica, PA = Panama, TT = Trnidad and Tobago, VE = Venezuela. Intermediate between *beryllina* and *cyanura*. * Specimens included in the analyses of microsatellite loci.

| **No.** | **Species phenotype** | **Museum** | **Voucher number** | **Locality** | **GeneBank accession numbers** | | **Microsatellites*** |
| --- | --- | --- | --- | --- | --- | --- | --- |
|  |  |  |  |  | **ND2** | **ATPase** |  |
| 1 | *A. beryllina* | UWBM | 82442 | MX: Sinaloa, Choix | KM198972 | KM199115 | X |
| 2 | *A. beryllina* | UWBM | 82488 | MX: Sinaloa, Choix | KM198973 | KM199116 | X |
| 3 | *A. beryllina* | UWBM | 82492 | MX: Sinaloa, Choix | KM198974 | KM199117 | X |
| 4 | *A. beryllina* | UWBM | 82751 | MX: Sinaloa, Choix | KM198975 | KM199118 | X |
| 5 | *A. beryllina* | UWBM | 82757 | MX: Sinaloa, Choix | KM198976 | KM199119 | X |
| 6 | *A. beryllina* | INECOL | VER155 | MX: Veracruz, Xalapa | KC858488 | KM199120 | X |
| 7 | *A. beryllina* | INECOL | VER202 | MX: Veracruz, Xalapa | KC858489 | KF640817 | X |
| 8 | *A. beryllina* | INECOL | VER172 | MX: Veracruz, Xalapa | KM198977 | KM199121 | X |
| 9 | *A. beryllina* | INECOL | VER197 | MX: Veracruz, Xalapa | KM198978 | KM199122 | X |
| 10 | *A. beryllina* | INECOL | VER205 | MX: Veracruz, Xalapa | KM198979 | KM199123 | X |
| 11 | *A. beryllina* | INECOL | Ab-Ris-1 | MX: Veracruz, Xalapa | KM198980 | KM199124 | X |
| 12 | *A. beryllina* | INECOL | Ab-Coap-1 | MX: Veracruz, Xalapa | KM198981 | KM199125 | X |
| 13 | *A. beryllina* | INECOL | Ab-43 | MX: Veracruz, Xalapa | KM198982 | KM199126 | X |
| 14 | *A. beryllina* | INECOL | Ab-25 | MX: Jalisco, Nevado de Colima | KM198983 | KM199127 | X |
| 15 | *A. beryllina* | INECOL | Ab-26 | MX: Jalisco, Nevado de Colima | KC858487 | KM199128 |  |
| 16 | *A. beryllina* | INECOL | Ab-41 | MX: Jalisco, San José de Gracia | KM198984 | KM199129 | X |
| 17 | *A. beryllina* | INECOL | Ab-42 | MX: Jalisco, San José de Gracia | KM198985 | KM199130 | X |
| 18 | *A. beryllina* | INECOL | Ab-37 | MX: Michoacán, Morelia | KM198986 | KM199131 | X |
| 19 | *A. beryllina* | INECOL | Ab-38 | MX: Michoacán, Morelia | KM198987 | KM199132 | X |
| 20 | *A. beryllina* | INECOL | Ab-39 | MX: Michoacán, Morelia | KM198988 | KM199133 | X |
| 21 | *A. beryllina* | INECOL | MX2708 | MX: DF, Pedregal | KM198989 | KM199134 | X |
| 22 | *A. beryllina* | INECOL | MX2726 | MX: DF, Pedregal | KM198990 | KM199135 | X |
| 23 | *A. beryllina* | INECOL | MX2618 | MX: DF, Pedregal | KM198991 | KM199136 | X |
| 24 | *A. beryllina* | INECOL | MX2836 | MX: DF, Pedregal | KM198992 | KM199137 | X |
| 25 | *A. beryllina* | INECOL | MX2639 | MX: DF, Pedregal | KM198993 | KM199138 | X |
| 26 | *A. beryllina* | INECOL | MX2838 | MX: DF, Pedregal | KM198994 | KM199139 | X |
| 27 | *A. beryllina* | INECOL | MX2842 | MX: DF, Pedregal | KM198995 | KM199140 | X |
| 28 | *A. beryllina* | INECOL | MX7148 | MX: DF, Pedregal | KM198996 | KM199141 | X |
| 29 | *A. beryllina* | UWBM | 115062 | MX: Guerrero, Omiltemi | KM198997 | KM199142 | X |
| 30 | *A. beryllina* | UWBM | 115063 | MX: Guerrero, Omiltemi | KM198998 | KM199143 | X |
| 31 | *A. beryllina* | UWBM | 115082 | MX: Guerrero, Omiltemi | KM198999 | KM199144 | X |
| 32 | *A. beryllina* | UWBM | 115083 | MX: Guerrero, Omiltemi | KM199000 | KM199145 | X |
| 33 | *A. beryllina* | UWBM | 115093 | MX: Guerrero, Omiltemi | KM199001 | KM199146 | X |
| 34 | *A. beryllina* | INECOL | Ab-SGM-1 | MX: Oaxaca, San Gabriel Mixtepec | KM199002 | KM199147 | X |
| 35 | *A. beryllina* | INECOL | Ab-SGM-2 | MX: Oaxaca, San Gabriel Mixtepec | KM199003 | KM199148 | X |
| 36 | *A. beryllina* | INECOL | Ab-SGM-3 | MX: Oaxaca, San Gabriel Mixtepec | KM199004 | KM199149 | X |
| 37 | *A. beryllina* | INECOL | Ab-SGM-4 | MX: Oaxaca, San Gabriel Mixtepec | KM199005 | KM199150 | X |
| 38 | *A. beryllina* | INECOL | Ab-PL-1 | MX: Oaxaca, Piedra Larga | KM199006 | KM199151 | X |
| 39 | *A. beryllina* | INECOL | Ab-40 | MX: Oaxaca, San Baltazar | KM199007 | KM199152 | X |
| 40 | *A. beryllina* | INECOL | Ab-CB-1 | MX: Oaxaca, Cerro Baúl | KC858486 | KM199153 | X |
| 41 | *A. beryllina* | INECOL | Ab-CB-3 | MX: Oaxaca, Cerro Baúl | KM199008 | KM199154 | X |
| 42 | *A. beryllina* | INECOL | Ab-CB-4 | MX: Oaxaca, Cerro Baúl | KM199009 | KM199155 | X |
| 43 | *A. beryllina* | INECOL | Ab-CB-5 | MX: Oaxaca, Cerro Baúl | KM199010 | KM199156 | X |
| 44 | *A. beryllina* | INECOL | Ab-CB-6 | MX: Oaxaca, Cerro Baúl | KM199011 | KM199157 | X |
| 45 | *A. beryllina* | INECOL | Ab-CB-7 | MX: Oaxaca, Cerro Baúl | KM199012 | KM199158 | X |
| 46 | *A. beryllina* | INECOL | Ab-17 | MX: Chiapas, Jitotol | KC858484 | KM199159 |  |
| 47 | *A. beryllina* | INECOL | Ab-15 | MX: Chiapas, Pueblo Nuevo | KC858485 | KM199160 |  |
| 48 | *A. beryllina* | INECOL | Ab-MS-1 | MX: Chiapas, Monte Sinaí | KM199013 | KM199161 | X |
| 49 | *A. beryllina* | INECOL | Ab-5 | MX: Chiapas, Salvador Urbina | KM199014 | KM199162 | X |
| 50 | *A. beryllina* | INECOL | Ab-6 | MX: Chiapas, Salvador Urbina | KM199015 | KM199163 | X |
| 51 | *A. beryllina* | INECOL | Ab-7 | MX: Chiapas, Salvador Urbina | KM199016 | KM199164 | X |
| 52 | *A. beryllina* | INECOL | Ab-8 | MX: Chiapas, Salvador Urbina | KM199017 | KM199165 | X |
| 53 | *A. beryllina* | INECOL | Ab-9 | MX: Chiapas, Salvador Urbina | KC858481 | KF640811 |  |
| 54 | *A. beryllina* | INECOL | Ab-18 | MX: Chiapas, Salvador Urbina | KM199018 | KM199166 | X |
| 55 | *A. beryllina* | INECOL | Ab-10 | MX: Chiapas, Nueva Colombia | KM199019 | KM199167 | X |
| 56 | *A. beryllina* | INECOL | Ab-11 | MX: Chiapas, Nueva Colombia | KM199020 | KM199168 | X |
| 57 | *A. beryllina* | INECOL | Ab-12 | MX: Chiapas, Nueva Colombia | KM199021 | KM199169 | X |
| 58 | *A. beryllina* | INECOL | Ab-13 | MX: Chiapas, Nueva Colombia | KM199022 | KM199170 | X |
| 59 | *A. beryllina* | INECOL | Ab-14 | MX: Chiapas, Nueva Colombia | KC858482 | KM199171 |  |
| 60 | *A. beryllina* | INECOL | Ab-19 | MX: Chiapas, Nueva Colombia | KC858483 | KM199172 |  |
| 61 | *A. beryllina* | INECOL | Ab-20 | MX: Chiapas, Nueva Colombia | KM199023 | KM199173 | X |
| 62 | *A. beryllina* | INECOL | Ab-21 | MX: Chiapas, Nueva Colombia | KM199024 | KM199174 | X |
| 63 | *A. beryllina* | INECOL | Ab-22 | MX: Chiapas, Nueva Colombia | KM199025 | KM199175 | X |
| 64 | *A. beryllina* | INECOL | Ab-23 | MX: Chiapas, Nueva Colombia | KM199026 | KM199176 | X |
| 65 | *A. beryllina* | INECOL | Ab-24 | MX: Chiapas, Nueva Colombia | KM199027 | KM199177 | X |
| 66 | *A. cyanura* | CZRAV | 6668 | MX: Chiapas, Escuintla | KM199046 | KM199196 |  |
| 67 | *A. cyanura* | CZRAV | 6517 | MX: Chiapas, Tapachula | KM199047 | KM199197 |  |
| 68 | *A. cyanura* | INECOL | Ac-16 | MX: Chiapas, Tapachula | KM199048 | KM199198 | X |
| 69 | *A. cyanura* | INECOL | Ac-17 | MX: Chiapas, Tapachula | KM199049 | KM199199 | X |
| 70 | *A. cyanura* | INECOL | Ac-18 | MX: Chiapas, Tapachula | KM199050 | KM199200 | X |
| 71 | *A. cyanura* | INECOL | Ac-19 | MX: Chiapas, Tapachula | KM199051 | KM199201 | X |
| 72 | *A. cyanura* | INECOL | Ac-20 | MX: Chiapas, Tapachula | KM199052 | KM199202 | X |
| 73 | *A. cyanura* | INECOL | Ac-12 (CHIS) | MX: Chiapas, Tacaná | KC858491 | KM199203 | X |
| 74 | *A. beryllina* | MUSHNAT | RAJ150 | GT: Huehuetenango, Todos Santos | KM199028 | KM199178 | X |
| 75 | *A. beryllina* | MUSHNAT | RAJ152 | GT: Huehuetenango, Todos Santos | KM199029 | KM199179 | X |
| 76 | *A. beryllina* | MUSHNAT | RAJ153 | GT: Huehuetenango, Todos Santos | KM199030 | KM199180 | X |
| 77 | *A. beryllina* | MUSHNAT | RAJ154 | GT: Huehuetenango, Todos Santos | KM199031 | KM199181 | X |
| 78 | *A. beryllina* | MUSHNAT | RAJ155 | GT: Huehuetenango, Todos Santos | KM199032 | KM199182 | X |
| 79 | *A. cyanura* | INECOL | Ac-15 | GT: San Marcos, El Tumbador | KM199053 | KM199204 | X |
| 80 | *A. cyanura* | INECOL | Ac-13 | GT: San Marcos, La Reforma | KM199054 | KM199205 | X |
| 81 | *A. cyanura* | INECOL | Ac-14 | GT: San Marcos, La Reforma | KM199055 | KM199206 | X |
| 82 | *A. cyanura* | MUSHNAT | RAJ156 | GT: Quetzaltenango, El Palmar | KM199056 | KM199207 | X |
| 83 | *A. cyanura* | MUSHNAT | RAJ157 | GT: Quetzaltenango, El Palmar | KM199057 | KM199208 | X |
| 84 | *A. cyanura* | MUSHNAT | RAJ158 | GT: Quetzaltenango, El Palmar | KF640749 | KF640816 | X |
| 85 | *A. cyanura* | MUSHNAT | RAJ159 | GT: Quetzaltenango, El Palmar | KM199058 | KM199209 | X |
| 86 | *A. cyanura* | MUSHNAT | RAJ160 | GT: Quetzaltenango, El Palmar | KM199059 | KM199210 | X |
| 87 | *A. cyanura* | MUSHNAT | RAJ161 | GT: Quetzaltenango, El Palmar | KM199060 | KM199211 | X |
| 88 | *A. beryllina* | MUSHNAT | RAJ115 | GT: Suchitepéquez, Patulul | KM199033 | KM199183 | X |
| 89 | *A. beryllina* | MUSHNAT | RAJ116 | GT: Suchitepéquez, Patulul | KM199034 | KM199184 | X |
| 90 | *A. beryllina* | MUSHNAT | RAJ134 | GT: Suchitepéquez, Patulul | KM199035 | KM199185 | X |
| 91 | *A. beryllina* | MUSHNAT | RAJ137 | GT: Suchitepéquez, Patulul | KM199036 | KM199186 | X |
| 92 | *A. cyanura* | MUSHNAT | RAJ129 | GT: Suchitepéquez, Patulul | KM199061 | KM199212 | X |
| 93 | *A. cyanura* | MUSHNAT | RAJ132 | GT: Suchitepéquez, Patulul | KM199062 | KM199213 | X |
| 94 | *A. cyanura* | MUSHNAT | RAJ133 | GT: Suchitepéquez, Patulul | KM199063 | KM199214 | X |
| 95 | Intermediate | MUSHNAT | RAJ111 | GT: Suchitepéquez, Patulul | KM199064 | KM199215 | X |
| 96 | Intermediate | MUSHNAT | RAJ112 | GT: Suchitepéquez, Patulul | KM199065 | KM199216 | X |
| 97 | Intermediate | MUSHNAT | RAJ113 | GT: Suchitepéquez, Patulul | KM199066 | KM199217 | X |
| 98 | Intermediate | MUSHNAT | RAJ124 | GT: Suchitepéquez, Patulul | KM199067 | KM199218 | X |
| 99 | Intermediate | MUSHNAT | RAJ127 | GT: Suchitepéquez, Patulul | KM199068 | KM199219 | X |
| 100 | Intermediate | MUSHNAT | RAJ128 | GT: Suchitepéquez, Patulul | KM199069 | KM199220 | X |
| 101 | Intermediate | MUSHNAT | RAJ130 | GT: Suchitepéquez, Patulul | KM199070 | KM199221 | X |
| 102 | Intermediate | MUSHNAT | RAJ131 | GT: Suchitepéquez, Patulul | KM199071 | KM199222 | X |
| 103 | Intermediate | MUSHNAT | RAJ136 | GT: Suchitepéquez, Patulul | KM199072 | KM199223 | X |
| 104 | Intermediate | MUSHNAT | RAJ140 | GT: Suchitepéquez, Patulul | KM199073 | KM199224 | X |
| 105 | Intermediate | INECOL | A-bc-1 | GT: Suchitepéquez, Patulul | KM199074 | KM199225 |  |
| 106 | Intermediate | INECOL | A-bc-2 | GT: Suchitepéquez, Patulul | KM199075 | KM199226 |  |
| 107 | Intermediate | INECOL | A-bc-3 | GT: Suchitepéquez, Patulul | KM199076 | KM199227 | X |
| 108 | Intermediate | INECOL | A-bc-4 | GT: Suchitepéquez, Patulul | KM199077 | KM199228 | X |
| 109 | Intermediate | INECOL | A-bc-5 | GT: Suchitepéquez, Patulul | KM199078 | KM199229 | X |
| 110 | Intermediate | INECOL | A-bc-8 | GT: Suchitepéquez, Patulul | KM199079 | KM199230 | X |
| 111 | Intermediate | INECOL | A-bc-9 | GT: Suchitepéquez, Patulul | KM199080 | KM199231 | X |
| 112 | *A. beryllina* | MUSHNAT | RAJ163 | GT: Chimaltenango | KM199037 | KM199187 | X |
| 113 | *A. beryllina* | MUSHNAT | RAJ164 | GT: Chimaltenango | KM199038 | KM199188 | X |
| 114 | Intermediate | MUSHNAT | RAJ162 | GT: Chimaltenango | KM199081 | KM199232 | X |
| 115 | Intermediate | MUSHNAT | RAJ165 | GT: Chimaltenango | KM199082 | KM199233 | X |
| 116 | *A. beryllina* | INECOL | Ab-30 | GT: Guatemala, Mixco | KM199039 | KM199189 | X |
| 117 | *A. beryllina* | INECOL | Ab-31 | GT: Guatemala, Mixco | KM199040 | KM199190 | X |
| 118 | *A. beryllina* | INECOL | Ab-35 | GT: Sacatepéquez, San Cristóbal | KM199041 | KM199191 | X |
| 119 | *A. beryllina* | INECOL | Ab-36 | GT: Sacatepéquez, San Cristóbal | KM199042 | KM199192 | X |
| 120 | *A. beryllina* | INECOL | Ab-32 | GT: Chiquimula, Esquipulas | KM199043 | KM199193 | X |
| 121 | *A. beryllina* | INECOL | Ab-33 | GT: Chiquimula, Esquipulas | KM199044 | KM199194 | X |
| 122 | *A. beryllina* | INECOL | Ab-34 | GT: Chiquimula, Esquipulas | KM199045 | KM199195 | X |
| 123 | *A. cyanura* | INECOL | Ac-1 | NIC: Jinotega, El Jaguar | KM199083 | KM199234 | X |
| 124 | *A. cyanura* | INECOL | Ac-2 | NIC: Jinotega, El Jaguar | KM199084 | KM199235 | X |
| 125 | *A. cyanura* | INECOL | Ac-3 | NIC: Jinotega, El Jaguar | KM199085 | KM199236 | X |
| 126 | *A. cyanura* | INECOL | Ac-4 | NIC: Jinotega, El Jaguar | KM199086 | KM199237 | X |
| 127 | *A. cyanura* | INECOL | Ac-5 | NIC: Jinotega, El Jaguar | KM199087 | KM199238 | X |
| 128 | *A. cyanura* | INECOL | Ac-6 | NIC: Jinotega, El Jaguar | KM199088 | KM199239 | X |
| 129 | *A. cyanura* | INECOL | Ac-7 | NIC: Jinotega, El Jaguar | KM199089 | KM199240 | X |
| 130 | *A. cyanura* | INECOL | Ac-8 | NIC: Jinotega, El Jaguar | KM199090 | KM199241 | X |
| 131 | *A. cyanura* | INECOL | Ac-9 | NIC: Jinotega, El Jaguar | KM199091 | KM199242 | X |
| 132 | *A. cyanura* | INECOL | Ac-10 | NIC: Jinotega, El Jaguar | KM199092 | KM199243 | X |
| 133 | *A. cyanura* | INECOL | Ac-11 | NIC: Jinotega, El Jaguar | KM199093 | KM199244 | X |
| 134 | *A. saucerottei* | INECOL | As-1 | NIC: Jinotega, El Jaguar | KM199094 | KM199245 | X |
| 135 | *A. saucerottei* | UWBM | 69093 | NIC: Granada | KM199095 | KM199246 | X |
| 136 | *A. saucerottei* | UWBM | 69107 | NIC: Granada | KM199096 | KM199247 | X |
| 137 | *A. saucerottei* | UWBM | 69336 | NIC: Granada | KM199097 | KM199248 | X |
| 138 | *A. saucerottei* | UWBM | 69337 | NIC: Granada | KM199098 | KM199249 | X |
| 139 | *A. saucerottei* | UWBM | 69338 | NIC: Granada | KM199099 | KM199250 | X |
| 140 | *A. saucerottei* | UWBM | 69380 | NIC: Granada | KM199100 | KM199251 | X |
| 141 | *A. saucerottei* | UWBM | 69389 | NIC: Granada | KM199101 | KM199252 | X |
| 142 | *A. saucerottei* | UWBM | 69394 | NIC: Granada | KM199102 | KM199253 | X |
| 143 | *A. saucerottei* | UWBM | 69395 | NIC: Granada | KM199103 | KM199254 | X |
| 144 | *A. saucerottei* | UWBM | 69409 | NIC: Granada | KM199104 | KM199255 | X |
| 145 | *A. saucerottei* | MCZ | 335513 | CR: Guanacaste | KM199105 | KM199256 | X |
| 146 | *A. saucerottei* | MCZ | 335577 | CR: Guanacaste | KM199106 | KM199257 | X |
| 147 | *A. saucerottei* | MCZ | 335581 | CR: Guanacaste | KM199107 | KM199258 | X |
| 148 | *A. saucerottei* | MCZ | 335591 | CR: Guanacaste | KM199108 | KM199259 | X |
| 149 | *A. saucerottei* | MCZ | 348244 | CR: Guanacaste | KM199109 | KM199260 | X |
| 150 | *A. saucerottei* | MCZ | 348245 | CR: Guanacaste | KM199110 | KM199261 | X |
| 151 | *A. saucerottei* | MCZ | 348248 | CR: Guanacaste | KM199111 | KM199262 | X |
| 152 | *A. saucerottei* | MCZ | 348249 | CR: Guanacaste | KM199112 | KM199263 | X |
| 153 | *A. saucerottei* | MCZ | 348453 | CR: Guanacaste | KM199113 | KM199264 | X |
| 154 | *A. saucerottei* | MCZ | 348454 | CR: Guanacaste | KM199114 | KM199265 | X |
| 155 | *A. saucerottei* | INECOL | VENE 149 | VE: Mérida | KC858492 | KF640818 |  |
| 156 | *A. saucerottei* | GenBank |  | VE: Venezuela | EU042523.1 | ----- |  |
| 157 | *A. saucerottei* | GenBank |  | CR: Costa Rica | GU167205.1 | ----- |  |
| 158 | *A. viridigaster* | GenBank |  |  | EU042526.1 | ----- |  |
| 159 | *A. viridigaster* | LSUMNS | 7587 | VE: Amazonas | KC858493 | KF640819 |  |
| 160 | *A. edward* | LSUMNS | 2303 | PA: Darien | KC858494 | KF640820 |  |
| 161 | *A. tobaci* | LSUMNS | B-35914 | TT: Trinidad and Tobago | KC858448 | KM199266 |  |
| 162 | *A. tobaci* | LSUMNS | B-69286 | TT: Trinidad and Tobago | KC858449 | KM199267 |  |
| 163 | *A. cyanocephala* | INECOL | G1 | GT: Chiquimula | KC858479 | KF640798 |  |
| 164 | *A. cyanocephala* | INECOL | G2 | GT: Chiquimula | KC858480 | KF640804 |  |
| 165 | *A. cyanocephala* | INECOL | Ord1 | MX: Veracruz, La Orduña | KC858469 | JX050062 |  |
| 166 | *A. cyanocephala* | INECOL | ATC4 | MX: Mexico, Tamaulipas | KC858471 | JX050061 |  |
| 167 | *A. cyanocephala* | INECOL | INE1 | MX: Veracruz, Xalapa | KC858470 | JX050060 |  |
| 168 | *A. violiceps* | INECOL | PUE129 | MX: Puebla | KC858513 | JX675222 |  |
| 169 | *A. viridifrons* | INECOL | PL1 | MX: Oaxaca, Piedra Larga | KC858516 | JX675223 |  |
| 170 | *Campylopterus curvipennis* | INECOL | 3-05 | MX: Veracruz, La Orduña | KC858427 | HQ380727 |  |
