# Supplementary material for: Historical and current introgression in a Mesoamerican hummingbird species complex: a biogeographic perspective
Source: PeerJ. 2016 Jan 12;4:e1556. doi: 10.7717/peerj.1556 (PMC4715438; doi:10.7717/peerj.1556)
Supplement: Supplemental Information 3 [file peerj-04-1556-s003.docx]

**Table S3** **Frequency and number of base pairs (bp), variable sites, and parsimony informative sites for the gene partitions, and DNA substitution models applied to each partition.**

| **Partition** | **Included bp** | **Frequency of variable sites**  **(no. variable sites)** | **Frequency of parsimony informative sites**  **(no. parsimony informative sites)** | **DNA substitution model** |
| --- | --- | --- | --- | --- |
| tRNA | 51 | 0.098 (5) | 0.039 (2) | K80 |
| ND2pos1 | 117 | 0.085 (10) | 0.043 (5) | HKY + G |
| ND2pos2 | 117 | 0.051 (6) | 0.034 (4) | HKY + I + G |
| ND2pos3 | 116 | 0.362 (42) | 0.201 (24) | HKY + G |
| ATPase6pos1 | 181 | 0.105 (19) | 0.072 (13) | HKY + G |
| ATPase6pos2 | 180 | 0.039 (7) | 0.028 (5) | HKY + I |
| ATPase6pos3 | 180 | 0.433 (78) | 0.289 (52) | HKY + G |
| ATPase8pos1 | 56 | 0.143 (8) | 0.072 (4) | HKY + G |
| ATPase8pos2 | 56 | 0.143 (8) | 0.054 (3) | HKY |
| ATPase8pos3 | 56 | 0.339 (19) | 0.196 (11) | HKY |
| TOTAL | 1110 | 0.182 (202) | 0.111 (123) |  |
