# Supplementary material for: Historical and current introgression in a Mesoamerican hummingbird species complex: a biogeographic perspective
Source: PeerJ. 2016 Jan 12;4:e1556. doi: 10.7717/peerj.1556 (PMC4715438; doi:10.7717/peerj.1556)
Supplement: Supplemental Information 4 [file peerj-04-1556-s004.docx]

**Table S4 Collection localities for the *Amazilia beryllina*, *A. cyanura*, *A. saucerottei* samples and those for individuals with intermediate phenotypes examined morphologically in this study**. *n* = sample size; MX = Mexico, GT = Guatemala, ES = El Salvador, NIC = Nicaragua, CR = Costa Rica. *Species phenotype according to current taxonomy: b = *beryllina*, c = *cyanura*, s = *saucerottei*, i = intermediate between *beryllina* and *cyanura*.

| **No.** | **Locality** | **Species phenotype*** | ***n*** | **Latitude**  **N** | **Longitude**  **W** | **Elevation**  **m asl** |
| --- | --- | --- | --- | --- | --- | --- |
| 1 | MX: Sinaloa, Mazatlán | b | 1 | 23.47602° | 105.83202° | 1740 |
| 2 | MX: Jalisco, Tuscueca | b | 2 | 20.15863° | 103.18389° | 1550 |
| 3 | MX: Michoacán, Tancitaro | b | 7 | 19.34415° | 102.36791° | 2050 |
| 4 | MX: Guerrero, Amojileca | b | 1 | 17.58257° | 99.57024° | 1900 |
| 5 | MX: Guerrero, Omiltemi | b | 1 | 17.54144° | 99.51715° | 1400 |
| 6 | MX: Guerrero, Cuapongo | b | 10 | 17.55533° | 99.50768° | 1250 |
| 7 | MX: Guerrero, Chilpancingo | b | 21 | 17.55245° | 99.50121° | 1270 |
| 8 | MX: DF, Ciudad de México | b | 2 | 19.43114° | 99.13373° | 2230 |
| 9 | MX: Morelos, Cuernavaca | b | 1 | 18.93069° | 99.09339° | 1370 |
| 10 | MX: Veracruz, Orizaba | b | 1 | 18.84627° | 97.10238° | 1240 |
| 11 | MX: Veracruz, Córdoba | b | 1 | 18.88391° | 96.92319° | 830 |
| 12 | MX: Veracruz, Motzorongo | b | 1 | 18.64313° | 96.73166° | 270 |
| 13 | MX: Tuxtla Gutiérrez, Montecristo | b | 1 | 16.72927° | 93.18867° | 660 |
| 14 | MX: Chiapas, Tuxtla Gutiérrez, El Zapotal | b | 2 | 16.73474° | 93.11067° | 590 |
| 15 | GT: Huehuetenango, Todos Santos Cuchumatán | b | 5 | 15.59093° | 91.68322° | 1800 |
| 16 | GT: Quetzaltenango, El Palmar | c | 6 | 14.66428° | 91.60941° | 750 |
| 17 | GT: Suchitepéquez, Mazatenango | c | 3 | 14.53477° | 91.50018° | 390 |
| 18 | GT: Suchitepéquez, Patulul | b, c, i | 21 | 14.54584° | 91.14759° | 1135 |
| 19 | GT: Chimaltenango, San Pedro Yepocapa | b, i | 3 | 14.44156° | 91.06678° | 690 |
| 20 | GT: Sacatepéquez, San Miguel Dueñas | b | 1 | 14.51676° | 90.79999° | 1500 |
| 21 | GT: Santa Rosa, Taxisco, El Cacahuito | b | 2 | 14.08986° | 90.44663° | 425 |
| 22 | GT: Zacapa, Usumatlán | b | 1 | 14.95003° | 89.77598° | 230 |
| 23 | ES: Ahuachapán, San Francisco Menéndez | b | 2 | 13.83380° | 89.93456° | 600 |
| 24 | ES: Ahuachapán, San Pedro Puxtla | b | 1 | 13.77117° | 89.80722° | 510 |
| 25 | ES: Sonsonate, Izalco, Las Lajas | b | 1 | 13.81649° | 89.58352° | 1125 |
| 26 | ES: Sonsonate, Chilata | b | 2 | 13.65514° | 89.53619° | 700 |
| 27 | ES: La Libertad, Santa Tecla | b | 1 | 13.68403° | 89.28317° | 935 |
| 28 | ES: La Libertad, San Salvador Volcano | b | 1 | 13.72560° | 89.23297° | 875 |
| 29 | ES: San Miguel Volcano | b | 2 | 13.43271° | 88.25513° | 1380 |
| 30 | ES: San Miguel | b | 3 | 13.47332° | 88.17660° | 125 |
| 31 | ES: San Miguel, Olomega Lake | b | 6 | 13.32779° | 88.06086° | 70 |
| 32 | NIC: Chinandega, San Jerónimo | c | 11 | 12.65000° | 87.21667° | 25 |
| 33 | NIC: Managua | s | 1 | 12.13705° | 86.24999° | 95 |
| 34 | NIC: Rivas, San Emilio, Nicaragua Lake | s | 2 | 11.18038° | 85.48636° | 50 |
| 35 | CR: Guanacaste | s | 4 | 10.45886° | 85.36046° | 100 |
| 36 | CR: Las Cañas | s | 2 | 10.42635° | 85.09593° | 90 |
| 37 | CR: San José | s | 5 | 9.92736° | 84.08214° | 1170 |
| 38 | CR: San Pedro | s | 1 | 9.95824° | 84.03909° | 1250 |
| 39 | CR: Cartago | s | 1 | 9.92580° | 83.98629° | 1400 |
| 40 | CR: Cartago, Irazú Volcano | s | 2 | 9.96530° | 83.85188° | 2900 |
| 41 | CR: Cartago, Orosi | s | 1 | 9.79332° | 83.84937° | 1070 |
| 42 | CR: Miravalles | s | 1 | 9.42217° | 83.67918° | 1080 |
| 43 | CR: Limón | s | 1 | 9.98946° | 83.03776° | 20 |
|  | TOTAL |  | 145 |  |  |  |
