# Supplementary material for: Historical and current introgression in a Mesoamerican hummingbird species complex: a biogeographic perspective
Source: PeerJ. 2016 Jan 12;4:e1556. doi: 10.7717/peerj.1556 (PMC4715438; doi:10.7717/peerj.1556)
Supplement: Supplemental Information 5 [file peerj-04-1556-s005.docx]

**Table S5** **Species names, voucher information, and localities of origin for the specimens examined morphologically in this study.** Specimens included are from the Museum of Vertebrate Zoology (MVZ), Field Museum of Natural History (FMNH) and Museo de Historia Natural, Universidad de San Carlos de Guatemala. Subspecies according to geography and current taxonomy (Dickinson & Remsen 2013: *Amazilia beryllina* (*viola*, *beryllina*, *lichtensteini*, *sumichrasti*, *devillei*), *Amazilia cyanura* (*guatemalae*, *cyanura*, *impatiens*), and *Amazilia saucerottei* (*hoffmanni*, *warscewiczi*, *saucerottei*, *braccata*). MX = Mexico, GT = Guatemala, ES = El Salvador, NIC = Nicaragua, CR = Costa Rica. Intermediate between *beryllina* and *cyanura*.

| **No.** | ***Subspecies phenotype** | **Museum** | **Voucher number** | **Locality** |
| --- | --- | --- | --- | --- |
|  |  |  |  |  |
| 1 | *A. b. viola* | MVZ | 116428 | MX: Sinaloa, Mazatlán |
| 2 | *A. b. viola* | FMNH | 124424 | MX: Jalisco, Tuscueca |
| 3 | *A. b. viola* | FMNH | 124426 | MX: Jalisco, Tuscueca |
| 4 | *A. b. viola* | FMNH | 102563 | MX: Michoacán, Tancitaro |
| 5 | *A. b. viola* | FMNH | 102564 | MX: Michoacán, Tancitaro |
| 6 | *A. b. viola* | FMNH | 102565 | MX: Michoacán, Tancitaro |
| 7 | *A. b. viola* | FMNH | 119101 | MX: Michoacán, Tancitaro |
| 8 | *A. b. viola* | FMNH | 119103 | MX: Michoacán, Tancitaro |
| 9 | *A. b. viola* | FMNH | 119104 | MX: Michoacán, Tancitaro |
| 10 | *A. b. viola* | FMNH | 119105 | MX: Michoacán, Tancitaro |
| 11 | *A. b. beryllina* | MVZ | 109935 | MX: Guerrero, Amojileca |
| 12 | *A. b. beryllina* | MVZ | 109937 | MX: Guerrero, Omiltemi |
| 13 | *A. b. beryllina* | MVZ | 109945 | MX: Guerrero, Cuapongo |
| 14 | *A. b. beryllina* | MVZ | 109946 | MX: Guerrero, Cuapongo |
| 15 | *A. b. beryllina* | MVZ | 109938 | MX: Guerrero, Cuapongo |
| 16 | *A. b. beryllina* | MVZ | 109939 | MX: Guerrero, Cuapongo |
| 17 | *A. b. beryllina* | MVZ | 109940 | MX: Guerrero, Cuapongo |
| 18 | *A. b. beryllina* | MVZ | 109941 | MX: Guerrero, Cuapongo |
| 19 | *A. b. beryllina* | MVZ | 109942 | MX: Guerrero, Cuapongo |
| 20 | *A. b. beryllina* | MVZ | 109947 | MX: Guerrero, Cuapongo |
| 21 | *A. b. beryllina* | MVZ | 109943 | MX: Guerrero, Cuapongo |
| 22 | *A. b. beryllina* | MVZ | 109944 | MX: Guerrero, Cuapongo |
| 23 | *A. b. beryllina* | MVZ | 109926 | MX: Guerrero, Chilpancingo |
| 24 | *A. b. beryllina* | MVZ | 109925 | MX: Guerrero, Chilpancingo |
| 25 | *A. b. beryllina* | MVZ | 109927 | MX: Guerrero, Chilpancingo |
| 26 | *A. b. beryllina* | MVZ | 109928 | MX: Guerrero, Chilpancingo |
| 27 | *A. b. beryllina* | MVZ | 109929 | MX: Guerrero, Chilpancingo |
| 28 | *A. b. beryllina* | MVZ | 109931 | MX: Guerrero, Chilpancingo |
| 29 | *A. b. beryllina* | MVZ | 109914 | MX: Guerrero, Chilpancingo |
| 30 | *A. b. beryllina* | MVZ | 109915 | MX: Guerrero, Chilpancingo |
| 31 | *A. b. beryllina* | MVZ | 109916 | MX: Guerrero, Chilpancingo |
| 32 | *A. b. beryllina* | MVZ | 109917 | MX: Guerrero, Chilpancingo |
| 33 | *A. b. beryllina* | MVZ | 109918 | MX: Guerrero, Chilpancingo |
| 34 | *A. b. beryllina* | MVZ | 109934 | MX: Guerrero, Chilpancingo |
| 35 | *A. b. beryllina* | MVZ | 109919 | MX: Guerrero, Chilpancingo |
| 36 | *A. b. beryllina* | MVZ | 109920 | MX: Guerrero, Chilpancingo |
| 37 | *A. b. beryllina* | MVZ | 109921 | MX: Guerrero, Chilpancingo |
| 38 | *A. b. beryllina* | MVZ | 109922 | MX: Guerrero, Chilpancingo |
| 39 | *A. b. beryllina* | MVZ | 109923 | MX: Guerrero, Chilpancingo |
| 40 | *A. b. beryllina* | MVZ | 109930 | MX: Guerrero, Chilpancingo |
| 41 | *A. b. beryllina* | MVZ | 109932 | MX: Guerrero, Chilpancingo |
| 42 | *A. b. beryllina* | MVZ | 109933 | MX: Guerrero, Chilpancingo |
| 43 | *A. b. beryllina* | MVZ | 109924 | MX: Guerrero, Chilpancingo |
| 44 | *A. b. beryllina* | FMNH | 302714 | MX: DF, Ciudad de México |
| 45 | *A. b. beryllina* | FMNH | 42400 | MX: DF, Ciudad de México |
| 46 | *A. b. beryllina* | MVZ | 153268 | MX: Morelos, Cuernavaca |
| 47 | *A. b. beryllina* | FMNH | 45460 | MX: Veracruz, Orizaba |
| 48 | *A. b. beryllina* | FMNH | 45466 | MX: Veracruz, Córdoba |
| 49 | *A. b. beryllina* | FMNH | 302713 | MX: Veracruz, Motzorongo |
| 50 | *A. b. devillei* | FMNH | 153206 | MX: Tuxtla Gutiérrez, Montecristo |
| 51 | *A. b. devillei* | FMNH | 153203 | MX: Chiapas, Tuxtla Gutiérrez, El Zapotal |
| 52 | *A. b. devillei* | FMNH | 153205 | MX: Chiapas, Tuxtla Gutiérrez, El Zapotal |
| 53 | *A. b. devillei* | MUSHNAT | RAJ150 | GT: Huehuetenango, Todos Santos Cuchumatán |
| 54 | *A. b. devillei* | MUSHNAT | RAJ152 | GT: Huehuetenango, Todos Santos Cuchumatán |
| 55 | *A. b. devillei* | MUSHNAT | RAJ153 | GT: Huehuetenango, Todos Santos Cuchumatán |
| 56 | *A. b. devillei* | MUSHNAT | RAJ154 | GT: Huehuetenango, Todos Santos Cuchumatán |
| 57 | *A. b. devillei* | MUSHNAT | RAJ155 | GT: Huehuetenango, Todos Santos Cuchumatán |
| 58 | *A. c. guatemalae* | MUSHNAT | RAJ156 | GT: Quetzaltenango, El Palmar |
| 59 | *A. c. guatemalae* | MUSHNAT | RAJ157 | GT: Quetzaltenango, El Palmar |
| 60 | *A. c. guatemalae* | MUSHNAT | RAJ158 | GT: Quetzaltenango, El Palmar |
| 61 | *A. c. guatemalae* | MUSHNAT | RAJ159 | GT: Quetzaltenango, El Palmar |
| 62 | *A. c. guatemalae* | MUSHNAT | RAJ160 | GT: Quetzaltenango, El Palmar |
| 63 | *A. c. guatemalae* | MUSHNAT | RAJ161 | GT: Quetzaltenango, El Palmar |
| 64 | *A. c. guatemalae* | FMNH | 22629 | GT: Suchitepéquez, Mazatenango |
| 65 | *A. c. guatemalae* | FMNH | 22631 | GT: Suchitepéquez, Mazatenango |
| 66 | *A. c. guatemalae* | FMNH | 22632 | GT: Suchitepéquez, Mazatenango |
| 67 | *A. b. devillei* | MUSHNAT | RAJ115 | GT: Suchitepéquez, Patulul |
| 68 | *A. b. devillei* | MUSHNAT | RAJ116 | GT: Suchitepéquez, Patulul |
| 69 | *A. b. devillei* | MUSHNAT | RAJ134 | GT: Suchitepéquez, Patulul |
| 70 | *A. b. devillei* | MUSHNAT | RAJ137 | GT: Suchitepéquez, Patulul |
| 71 | *A. b. devillei* | FMNH | 22626 | GT: Suchitepéquez, Patulul |
| 72 | *A. b. devillei* | FMNH | 22628 | GT: Suchitepéquez, Patulul |
| 73 | *A. b. devillei* | FMNH | 22627 | GT: Suchitepéquez, Patulul |
| 74 | *A. c. guatemalae* | FMNH | 22634 | GT: Suchitepéquez, Patulul |
| 75 | *A. c. guatemalae* | MUSHNAT | RAJ129 | GT: Suchitepéquez, Patulul |
| 76 | *A. c. guatemalae* | MUSHNAT | RAJ132 | GT: Suchitepéquez, Patulul |
| 77 | *A. c. guatemalae* | MUSHNAT | RAJ133 | GT: Suchitepéquez, Patulul |
| 78 | Intermediate | MUSHNAT | RAJ111 | GT: Suchitepéquez, Patulul |
| 79 | Intermediate | MUSHNAT | RAJ112 | GT: Suchitepéquez, Patulul |
| 80 | Intermediate | MUSHNAT | RAJ113 | GT: Suchitepéquez, Patulul |
| 81 | Intermediate | MUSHNAT | RAJ124 | GT: Suchitepéquez, Patulul |
| 82 | Intermediate | MUSHNAT | RAJ127 | GT: Suchitepéquez, Patulul |
| 83 | Intermediate | MUSHNAT | RAJ128 | GT: Suchitepéquez, Patulul |
| 84 | Intermediate | MUSHNAT | RAJ130 | GT: Suchitepéquez, Patulul |
| 85 | Intermediate | MUSHNAT | RAJ131 | GT: Suchitepéquez, Patulul |
| 86 | Intermediate | MUSHNAT | RAJ136 | GT: Suchitepéquez, Patulul |
| 87 | Intermediate | MUSHNAT | RAJ140 | GT: Suchitepéquez, Patulul |
| 88 | *A. beryllina* | MUSHNAT | RAJ163 | GT: Chimaltenango, San Pedro Yepocapa |
| 89 | *A. beryllina* | MUSHNAT | RAJ164 | GT: Chimaltenango, San Pedro Yepocapa |
| 90 | Intermediate | MUSHNAT | RAJ165 | GT: Chimaltenango, San Pedro Yepocapa |
| 91 | *A. b. devillei* | FMNH | 42401 | GT: Sacatepéquez, San Miguel Dueñas |
| 92 | *A. b. devillei* | FMNH | 212768 | GT: Santa Rosa, Taxisco, El Cacahuito |
| 93 | *A. b. devillei* | FMNH | 212770 | GT: Santa Rosa, Taxisco, El Cacahuito |
| 94 | *A. b. devillei* | FMNH | 212769 | GT: Zacapa, Usumatlán |
| 95 | *A. b. devillei* | FMNH | 433997 | ES: Ahuachapán, San Francisco Menéndez |
| 96 | *A. b. devillei* | FMNH | 433999 | ES: Ahuachapán, San Francisco Menéndez |
| 97 | *A. b. devillei* | FMNH | 433993 | ES: Ahuachapán, San Pedro Puxtla |
| 98 | *A. b. devillei* | FMNH | 433995 | ES: Sonsonate, Izalco, Las Lajas |
| 99 | *A. b. devillei* | MVZ | 85754 | ES: Sonsonate, Chilata |
| 100 | *A. b. devillei* | MVZ | 81280 | ES: Sonsonate, Chilata |
| 101 | *A. b. devillei* | FMNH | 212262 | ES: La Libertad, Santa Tecla |
| 102 | *A. b. devillei* | FMNH | 212263 | ES: La Libertad, San Salvador Volcano |
| 103 | *A. b. devillei* | FMNH | 111386 | ES: San Miguel Volcano |
| 104 | *A. b. devillei* | FMNH | 111387 | ES: San Miguel Volcano |
| 105 | *A. b. devillei* | FMNH | 111382 | ES: San Miguel |
| 106 | *A. b. devillei* | FMNH | 111384 | ES: San Miguel |
| 107 | *A. b. devillei* | FMNH | 111385 | ES: San Miguel |
| 108 | *A. b. devillei* | FMNH | 111389 | ES: San Miguel, Olomega Lake |
| 109 | *A. b. devillei* | FMNH | 111388 | ES: San Miguel, Olomega Lake |
| 110 | *A. b. devillei* | FMNH | 111390 | ES: San Miguel, Olomega Lake |
| 111 | *A. b. devillei* | MVZ | 85749 | ES: San Miguel, Olomega Lake |
| 112 | *A. b. devillei* | MVZ | 85752 | ES: San Miguel, Olomega Lake |
| 113 | *A. b. devillei* | MVZ | 85751 | ES: San Miguel, Olomega Lake |
| 114 | *A. c. cyanura* | FMNH | 15614 | NIC: Chinandega, San Jerónimo |
| 115 | *A. c. cyanura* | FMNH | 15618 | NIC: Chinandega, San Jerónimo |
| 116 | *A. c. cyanura* | FMNH | 15619 | NIC: Chinandega, San Jerónimo |
| 117 | *A. c. cyanura* | FMNH | 15620 | NIC: Chinandega, San Jerónimo |
| 118 | *A. c. cyanura* | FMNH | 15621 | NIC: Chinandega, San Jerónimo |
| 119 | *A. c. cyanura* | FMNH | 15622 | NIC: Chinandega, San Jerónimo |
| 120 | *A. c. cyanura* | FMNH | 15623 | NIC: Chinandega, San Jerónimo |
| 121 | *A. c. cyanura* | FMNH | 15625 | NIC: Chinandega, San Jerónimo |
| 122 | *A. c. cyanura* | FMNH | 15627 | NIC: Chinandega, San Jerónimo |
| 123 | *A. c. cyanura* | FMNH | 15628 | NIC: Chinandega, San Jerónimo |
| 124 | *A. c. cyanura* | FMNH | 15629 | NIC: Chinandega, San Jerónimo |
| 125 | *A. s. hoffmanni* | FMNH | 372381 | NIC: Managua |
| 126 | *A. s. hoffmanni* | FMNH | 21923 | NIC: Rivas, San Emilio, Nicaragua Lake |
| 127 | *A. s. hoffmanni* | FMNH | 21924 | NIC: Rivas, San Emilio, Nicaragua Lake |
| 128 | *A. s. hoffmanni* | MVZ | 157119 | CR: Guanacaste |
| 129 | *A. s. hoffmanni* | MVZ | 157120 | CR: Guanacaste |
| 130 | *A. s. hoffmanni* | MVZ | 157121 | CR: Guanacaste |
| 131 | *A. s. hoffmanni* | MVZ | 157122 | CR: Guanacaste |
| 132 | *A. s. hoffmanni* | FMNH | 72104 | CR: Las Cañas |
| 133 | *A. s. hoffmanni* | FMNH | 72105 | CR: Las Cañas |
| 134 | *A. s. hoffmanni* | FMNH | 6796 | CR: San José |
| 135 | *A. s. hoffmanni* | FMNH | 6797 | CR: San José |
| 136 | *A. s. hoffmanni* | FMNH | 45878 | CR: San José |
| 137 | *A. s. hoffmanni* | FMNH | 45879 | CR: San José |
| 138 | *A. s. hoffmanni* | FMNH | 302715 | CR: San José |
| 139 | *A. s. hoffmanni* | MVZ | 54045 | CR: San Pedro |
| 140 | *A. s. hoffmanni* | MVZ | 157118 | CR: Cartago |
| 141 | *A. s. hoffmanni* | FMNH | 72106 | CR: Cartago, Irazú Volcano |
| 142 | *A. s. hoffmanni* | FMNH | 72107 | CR: Cartago, Irazú Volcano |
| 143 | *A. s. hoffmanni* | FMNH | 21925 | CR: Cartago, Orosi |
| 144 | *A. s. hoffmanni* | FMNH | 72103 | CR: Miravalles |
| 145 | *A. s. hoffmanni* | FMNH | 45880 | CR: Limón |
