# Supplementary material for: Historical and current introgression in a Mesoamerican hummingbird species complex: a biogeographic perspective
Source: PeerJ. 2016 Jan 12;4:e1556. doi: 10.7717/peerj.1556 (PMC4715438; doi:10.7717/peerj.1556)
Supplement: Supplemental Information 6 [file peerj-04-1556-s006.docx]

**Table S6 Colour characters and index scores used to assess plumage colour variation.**

| **No.** | **Colour character** | **Score** |
| --- | --- | --- |
| 1 | Rufous patch on secondary feathers |  |
|  | Present and in > 50% as in *A. beryllina* | 3 |
|  | Present and between 10 and 40% in 6 feathers | 2 |
|  | Present and between 10 and 60% in < 6 feathers | 1 |
|  | Absent as in *A. saucerottei* | 0 |
| 2 | Rufous patch on primary feathers |  |
|  | Present in 10 feathers as in *A. beryllina* | 2 |
|  | Present in 3 to 7 feathers | 1 |
|  | Absent as in *A. saucerottei* | 0 |
| 3 | Undertail feathers |  |
|  | Rufous brown with white edge as in *A. beryllina* | 5 |
|  | Rufous brown with/without violet shades | 4 |
|  | Rufous-grey | 3 |
|  | Purple | 2 |
|  | Blue | 1 |
|  | Blue-grey as in *A. saucerottei* | 0 |
| 4 | Belly colour |  |
|  | Greyish brown as in *A. beryllina* | 1 |
|  | Emerald green with/without grey as in *A. saucerottei* | 0 |
| 5 | Throat and chest colour |  |
|  | Golden-green as in *A. beryllina* | 2 |
|  | Green, between gold and emerald | 1 |
|  | Emerald-green as in *A. saucerottei* | 0 |
| 6 | Wings |  |
|  | Smoked black as in *A. beryllina* | 1 |
|  | Bluish black as in *A. saucerottei* | 0 |
| 7 | Uppertail feathers |  |
|  | Violet as in *A. beryllina* | 4 |
|  | Rufous-brown | 3 |
|  | Purple | 2 |
|  | Purple-blue | 1 |
|  | Blue as in *A. saucerottei* | 0 |
| 8 | Back colour |  |
|  | Golden-green as in *A. beryllina* | 2 |
|  | Green, between gold and emerald | 1 |
|  | Emerald-green as in *A. saucerottei* | 0 |
| 9 | Tail colour |  |
|  | Rufous brown with violet shades as in *A. beryllina* | 3 |
|  | Rufous brown with purple | 2 |
|  | Purple-blue | 1 |
|  | Blue as in *A. saucerottei* | 0 |
| 10 | Rachis colour on tail feathers |  |
|  | Rufous (T1 can be black) as in *A. beryllina* | 1 |
|  | Black as in *A. saucerottei* | 0 |
