# Supplementary material for: Historical and current introgression in a Mesoamerican hummingbird species complex: a biogeographic perspective
Source: PeerJ. 2016 Jan 12;4:e1556. doi: 10.7717/peerj.1556 (PMC4715438; doi:10.7717/peerj.1556)
Supplement: Supplemental Information 7 [file peerj-04-1556-s007.docx]

**Table S7** **Summary statistics, neutrality tests and population expansion tests for *Amazilia beryllina*, *A. cyanura* and *A. saucerottei*.** Number of individuals sequenced (*n*), number of haplotypes (*H*), number of segregating sites (*S*), *h* = gene diversity, *π* = nucleotide diversity, *D* = Tajima’s *D*, *F_S_* = Fu and Li’s *F*s, SDD = differences in the sum of squares or mismatch distribution, Hri = Harpending’s raggedness index for mtDNA. Positive values for *D* and *F_S_* are indicative of mutation-drift-equilibrium, which is typical of stable populations, whereas negative values that result from an excess of rare haplotypes, indicate that populations have undergone recent expansions, often preceded by a bottleneck. Significantly negative values (at the *P* = 0.05 level for Tajima’s *D* and for *Fs* tests; Excoffier and Lischer 2010) in both tests reveal historic demographic expansion events. Significant (*P* ≤ 0.05) SSD and Hri values indicate deviations from the sudden expansion model. Values that are consistent with demographic expansion are shown in bold. **P* < 0.05.

| **Locus** | **Species** | ***n*** | ***H*** | ***S*** | ***h*** | ***π*** | **Tajima’s *D*** | | **Fu and Li’s *Fs*** | **SSD** | **Hri** |
| --- | --- | --- | --- | --- | --- | --- | --- | --- | --- | --- | --- |
| *ND2* | *A. beryllina* | 39 | 8 | 12 | 0.699±0.067 | 0.0084±0.001 | | 0.0954 | 0.4622 | 0.0622 | 0.1315 |
|  | *A. cyanura* | 95 | 18 | 20 | 0.897±0.015 | 0.0104±0.001 | | –0.1916 | –0.0249 | 0.0064 | 0.0105 |
|  | *A. saucerottei* | 20 | 7 | 11 | 0.774±0.065 | 0.0087±0.001 | | –0.0608 | –0.7645 | 0.0662 | 0.1297 |
| *ATPase* | *A. beryllina* | 39 | 16 | 14 | 0.916±0.023 | 0.0045±0.0004 | | –0.3924 | 0.6310 | **0.0609*** | 0.0219 |
|  | *A. cyanura* | 95 | 23 | 27 | 0.881±0.019 | 0.0038±0.0002 | | –1.5377 | **–2.4463*** | 0.0001 | 0.0109 |
|  | *A. saucerottei* | 20 | 5 | 12 | 0.726±0.062 | 0.0065±0.0004 | | 1.5491 | 0.6403 | **0.1311*** | 0.2637 |
| *ND2* + *ATPase* | *A beryllina* | 39 | 19 | 26 | 0.941±0.018 | 0.0059±0.006 | –0.1789 | | 0.6381 | 0.0209 | 0.0170 |
|  | *A. cyanura* | 95 | 32 | 47 | 0.933±0.014 | 0.0061±0.009 | ­–1.0378 | | –1.6358 | 0.0105 | 0.0127 |
|  | *A. saucerottei* | 20 | 11 | 23 | 0.868±0.057 | 0.0072±0.006 | 0.8342 | | ­–0.0415 | 0.0416 | 0.0660 |
