# Supplementary figures and images for: Historical and current introgression in a Mesoamerican hummingbird species complex: a biogeographic perspective
Source: PeerJ. 2016 Jan 12;4:e1556. doi: 10.7717/peerj.1556 (PMC4715438; doi:10.7717/peerj.1556)

■ *Amazilia beryllina*  
■ *Amazilia cyanura*  
■ *Amazilia saucerrottei*

0.007 substitutions/site

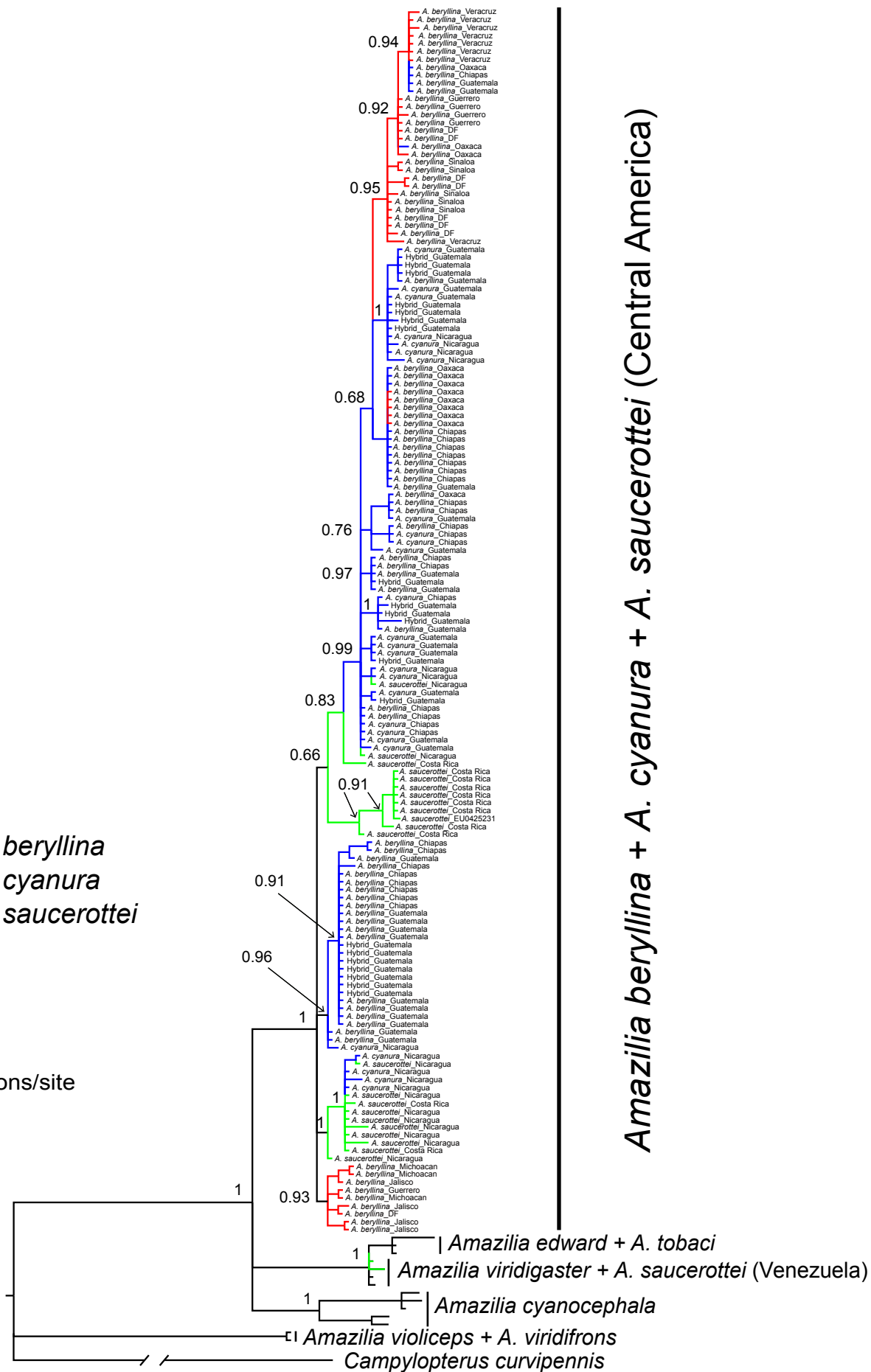

Supplement: Supplemental Information 8 [file peerj-04-1556-s008.pdf]

A

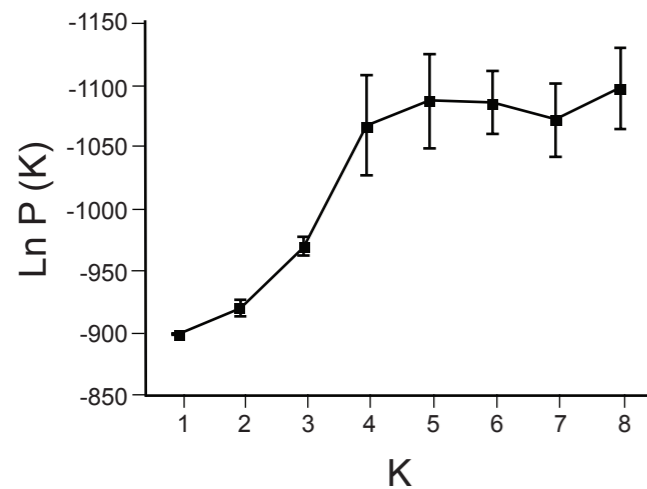

B

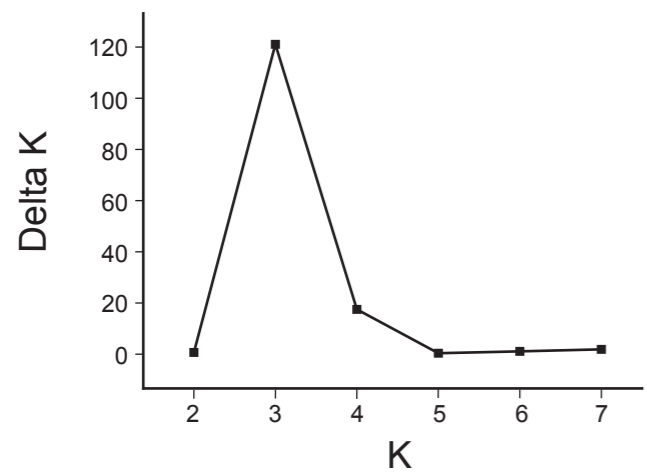

C

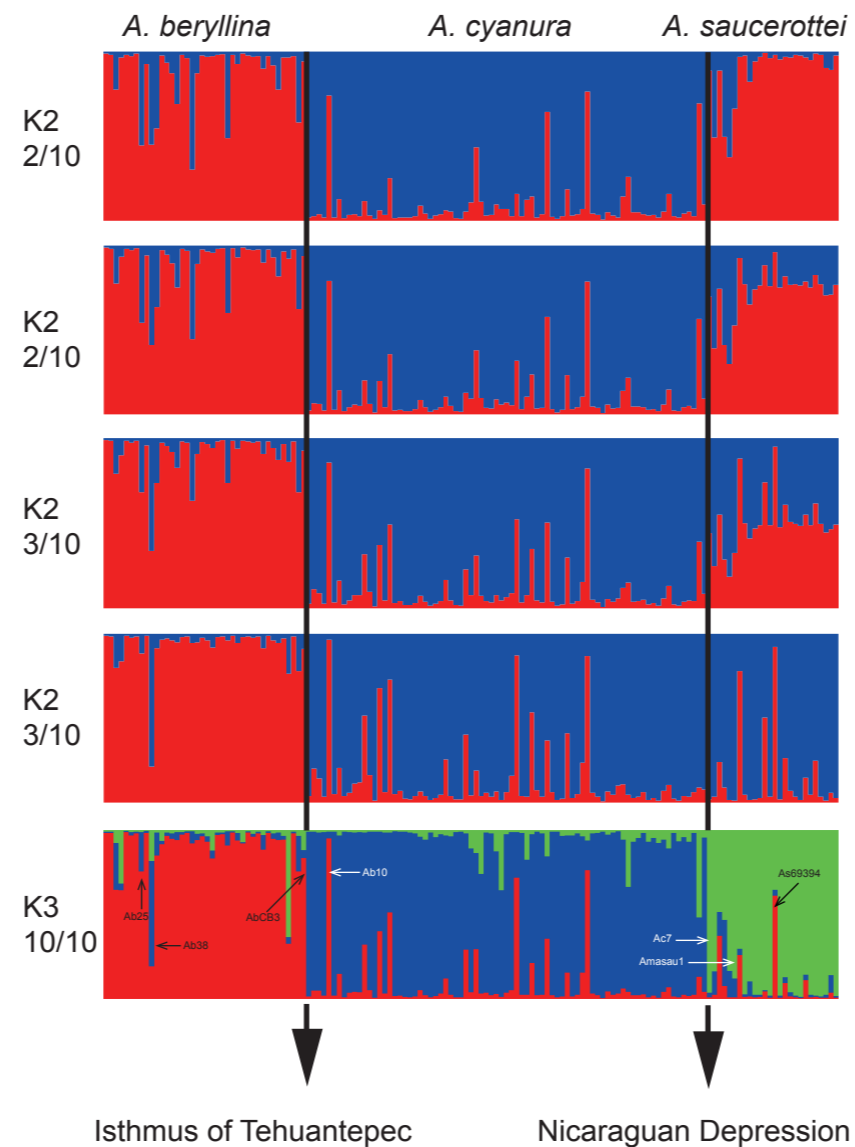

D

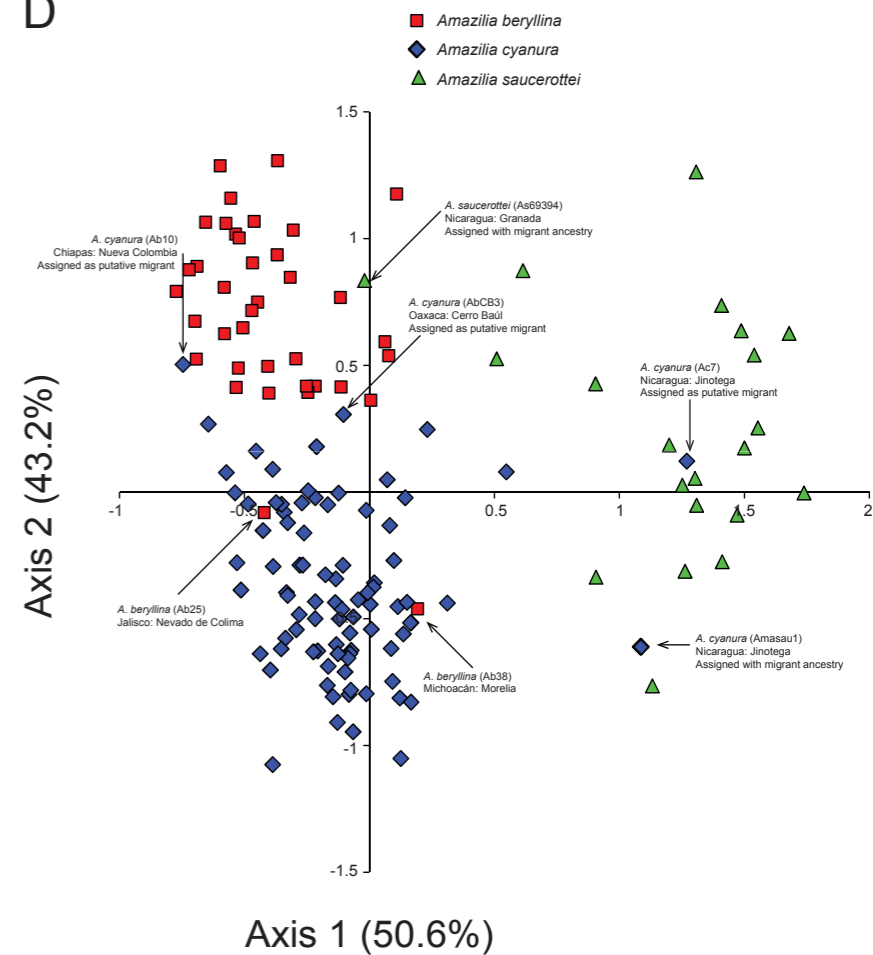

Supplement: Supplemental Information 9 [file peerj-04-1556-s009.pdf]

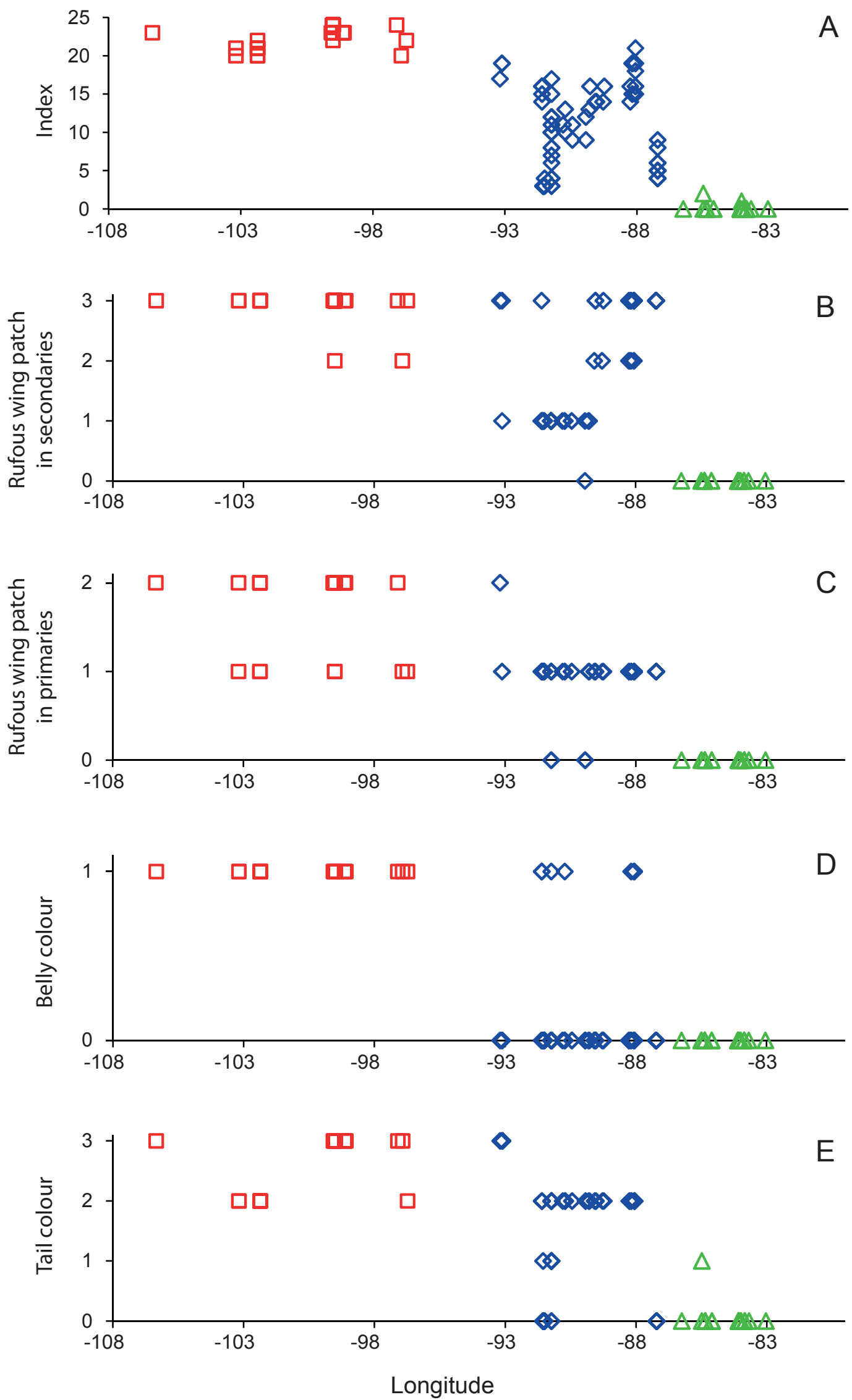

Supplement: Supplemental Information 10 [file peerj-04-1556-s010.pdf]

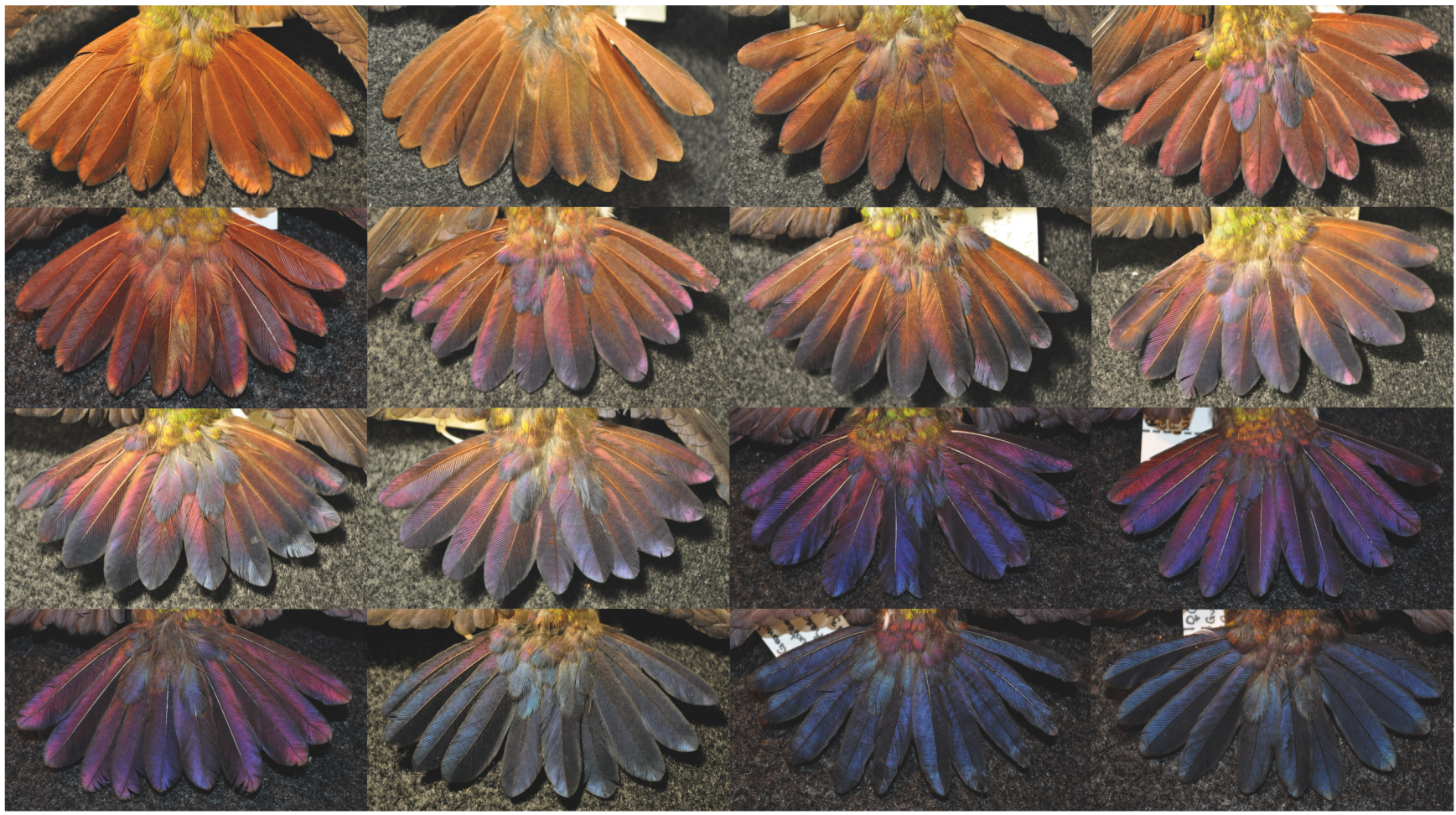

Supplement: Supplemental Information 11 — Figure 4: Present and past distribution models and environmental space for Amazilia beryllina, A. cyanura, and A. saucerottei. (A) Species distribution models generated with MaxEnt v. 3.3.3k for beryllina, cyanura and saucerottei for the present, Last Glacial Maximum (LGM, MIROC), Last Glacial Maximum (LGM, CCSM), and Last Interglacial (LIG). Darker shading indicates the most probable predicted distribution. (B) Principal components analysis showing occurrence points (closed symbols) and 1000 random points from the background (open symbols) where each of the three species is found; beryllina (red squares), cyanura (blue diamonds), and saucerottei (green triangles). [file peerj-04-1556-s011.pdf]
